# Polyphenolic profile, hepatoprotective evaluation, and molecular docking study of three palm tree species (Family Arecaceae)

**DOI:** 10.1007/s44446-025-00017-3

**Published:** 2025-07-08

**Authors:** Fadila M. Hamed, Heba E. Elsayed, Mohamed S. Mady, Merhan E. Ali, Asmaa A. Ahmed, Sabah H. Elgayed, Doaa Abouelenein, Giovanni Caprioli, Yara E. Mansour, Ahmed M. Mustafa, Elsayed K. El-Sayed, Fatma A. Moharram

**Affiliations:** 1 Department of Pharmacognosy, Faculty of Pharmacy, October 6 University, Giza, 12585 Egypt; 2 Department of Pharmacognosy, Faculty of Pharmacy, Helwan University, Cairo, 11795 Egypt; 3 Department of Cytology and Histology, Faculty of Veterinary Medicine, Cairo University, Giza, 12211 Egypt; 4 Department of Pharmacology and Toxicology, Faculty of Pharmacy, Helwan University, Cairo, 11795 Egypt; 5 Department of Pharmacognosy, Faculty of Pharmacy, Cairo University, Cairo, 11562 Egypt; 6 School of Pharmacy, University of Camerino, Via Sant’ Agostino 1, 62032 Camerino, Italy; 7 Department of Pharmaceutical Organic Chemistry, Faculty of Pharmacy, Helwan University, Cairo, 11795 Egypt; 8 Department of Pharmacognosy, Faculty of Pharmacy, Zagazig University, Zagazig, Egypt

**Keywords:** *Aiphanes eggersii*, *Carpoxylon macrospermum*, Hepatoprotective, *Jubaeopsis caffra*, HPLC–MS/MS, Molecular docking

## Abstract

Arecaceae species are renowned in traditional medicine for treating inflammation and liver disorders. Herein, we aimed to identify the phenolic constituents and the hepatoprotective potential of the aqueous methanol extract (AME) of *Aiphanes eggersii, Carpoxylon macrospermum*, and *Jubaeopsis caffra* leaves, in a drug-induced liver injury *in vivo* model. The AMEs are considered safe until the maximum tested dose (5 g/kg). The two selected screening doses, 500 and 1000 mg/kg, displayed antioxidant activity with significant (*P* < 0.05) decline in the liver/body weight ratios (19.1–29.7%), liver enzymes (25.9–63.4%), and malondialdehyde (39.3–63.8%), while increasing reduced glutathione (2.1–3.2 folds) and superoxide dismutase (2.2–3.1 folds). Moreover, they demonstrated a significant anti-inflammatory effect (*P* < 0.05) with decline in NF-_K_B p65 (32.7–64.5%), tumor necrosis factor-alpha (24.9–64.4%), and interleukin-1β (18.7–64.2%). Ultimately, significant (*P* < 0.05) antiapoptotic effects from the declined BAX (31.8–65.6%) and caspase-3 (23–69%), while increasing Bcl2 (2.7–5.7 folds). Ultimately, the histopathological investigation showed obvious hepatoprotective efficacy. The HPLC–MS/MS profiling revealed high phenolic content. As key phenolic attributes, chlorogenic acid is major in *C. macrospermum* and *J. caffra,* while vanillic in *A. eggersii*. Rutin is the principal flavonol in the three extracts (365.852–57970.205 μg/Kg), followed by hyperoside (62.764–7379.297 μg/Kg) and hesperidin (1225.976–1575.550 μg/Kg). The docking results show that rutin and hesperidin achieved the best fitting to SOD-1, with binding scores of -8.24 and -8.36 kcal/mol, while -8.0671 and -7.1735 kcal/mol with caspase-3, respectively with stable conformations revealed by 100 ns MD. In all, the investigated species exert significant hepatoprotective activity, at least partly, to their constitutive flavonoids and phenolic acids. However, further clinical investigation is still needed.

**Supplementary Information:**

The online version contains supplementary material available at 10.1007/s44446-025-00017-3.

## Introduction

Drug-induced liver injury (DILI) is a potentially lethal source of liver hepatotoxicity caused by natural or synthetic medications (Li et al. 2022). DILI accounts for less than 1% of acute liver injury (ALI) and 10–50% acute liver failure mortality (FDA United States Food and Drug Administration 2009). It has been associated with nearly 1,000 medications; hence, it is the leading cause of global drug withdrawal (Kullak-Ublick et al. 2017). Paracetamol (PCM, acetaminophen) is a common over-the-counter drug that induces intrinsic hepatotoxicity that often metamorphoses into hepatitis, cirrhosis, or even cancer (Kaplowitz 2004; Tafere et al. 2020). At therapeutic levels, PCM is metabolized in the liver by conjugation with glucuronic acid and cytochrome P450 (CYP450) into N-acetyl-*p*-benzoquinoimine (NAPQI). Subsequently, NAPQI is rapidly detoxified by reduced glutathione (GSH) (Mazaleuskaya et al. 2015). PCM overdosing causes depletion of GSH and binding of NAPQI to mitochondrial proteins resulting in necrosis (Xu et al. 2017; Woolbright and Jaeschke 2015). Meanwhile, liver mitochondrial enzymes such as alanine aminotransferase (ALT) and aspartate aminotransferase (AST) are upregulated and considered diagnostic features for DILI (Contreras-Zentella and Hernández-Muñoz 2016). The exact mechanisms involved in hepatocyte necrosis are not fully understood. However, covalent bond formation, lipid peroxidation, oxidative stress, inflammation, and apoptosis coincided in injured hepatocytes (Galal et al. 2012). Despite the vast advances in modern medicine, most documented therapies lack consistency and display profound side effects (Lee and Senior 2005). Urging researchers to explore alternative treatments with supreme efficacy and minimal adverse effects.

The global paradigm is shifting towards the therapeutic evaluation of herbal medicines to control liver disorders. Because herbal-based therapies are characterized by relative convenience, safety, and efficacy. Many researchers have presented reviews on the hepatoprotective potential of natural products and phytochemicals frequently consumed by humans (Ilyas et al. 2014; Madrigal-Santillán et al. 2014; Ali et al. 2018; Gillessen et al. 2022; Arman et al. 2022). However, some lack scientific evidence, while others still have room to augment their ethnopharmacological significance. Hence, unraveling the underlying hepatoprotective mechanism of traditionally used natural products is a promising strategy to support protection against DILI.

Arecaceae is a large family of perennial, flowering plants with the majority being well-known as palm trees (Emilio et al. 2019). The family includes about 2600 species and 181 genera, allocated in the tropical and subtropical regions (Emilio et al. 2019). Due to their therapeutic merits, African palm trees have been adopted as folk medicine for treating and controlling several disorders. Examples are, but not limited to, inflammation, liver diseases, candidiasis, headaches, and diarrhea (Gruca et al. 2015; Syahmi et al. 2010). Palm trees are prolific with diverse phytoconstituents such as flavonoids, tannins, phenolic acids, steroids, alkaloids, carotenoids, and lignans (Mohammed and Fouad 2022). These phytochemicals possess various pharmacological activities, including anti-inflammatory, and hepatoprotective (Mohammed and Fouad 2022). The heterogeneity of the phytochemical constituents and their concurrent plethora of biological significance were previously reported on palm trees. Thus, according to the hepatoprotective relevance of Arecaceae species, and our research interest in the discovery of effective and safe hepatoprotective remedy inspired by natural products, herein we address for the first time the hepatoprotective potential of the aqueous methanol extract of three unexplored palm tree species, *Aiphanes eggersii* Burret, *Carpoxylon macrospermum* H.Wendl. & Drude, and *Jubaeopsis caffra* Becc. in a paracetamol-induced liver hepatotoxicity animal model. Concurrently, hyphenated HPLC–MS/MS was implemented to investigate their phenolic profile, while molecular docking and molecular dynamics correlated their significant hepatoprotective activity with the phytochemical composition.

## Materials and methods

### Plant material

The leaves of the three palm tree species, *Aiphanes eggersii* Burret, *Carpoxylon macrospermum* H.Wendl. & Drude, and *Jubaeopsis caffra* Becc. were obtained from El Abd Garden, Cairo-Alexandria Desert Road, Egypt, in June 2022 after the endorsement of the local garden`s guidelines, and the collection rules of Egypt. All species were botanically authenticated by Dr. Trease Labib, Senior Botanist at Mazhar Botanical Garden, Cairo, Egypt, and assigned as 01 Aeg/2022, 01 Cma/2022, and 01 Jca/2022, respectively.

### Extraction of the phenolic constituents

About 0.5 kg of the air-dried leaves of each species were extracted with 80% aqueous methanol (5 × 1.5 L) under reflux at 70 °C for 4 h. Thereafter, the extracts were filtered and dried under vacuum at 70 °C using a rotary evaporator (Truong et al. 2019; Elsayed et al. 2022). A sample from each extract was investigated using two-dimensional (2D) chromatography eluted with butanol: acetic acid: water (BAW, 4:1:5, upper layer), followed by 15% acetic acid/water (AcOH). The dried 2D-PC was sprayed with 1% ferric chloride, 1% aluminum chloride, and Naturstoff (Elsayed et al. 2022). The remaining extracts were stored in pre-weighted amber glass vials at −20 °C for the HPLC analysis and the in vivo study.

### Phenolic profiling using HPLC–MS/MS

#### Reagents and standards

Kaempferol- and quercetin-3-*O*-glucoside analytical standards were supplied by the Phyto Lab GmbH & Co. KG (Vestenbergsgreuth, Germany), while the remaining 36 standard phenolics were purchased from Sigma-Aldrich (Milan, Italy). 99% formic acid was purchased from Merck (Darmstadt, Germany). Milli-Q SP Reagent Water System filtered deionized water to give ultrapure water with a sensitivity of > 18 M cm (Millipore, Bedford, MA, USA). Polyamide filters (0.2 μm, Sartorius Stedim, Goettingen, Germany) were used for sample filtration before usage.

#### Preparation of stock solutions

Standards'stock solutions (1 g/L) were first prepared in HPLC-grade MeOH (Sigma-Aldrich, Milan, Italy). Different concentrations were obtained by serially diluting each stock, followed by filtration on Phenex™ RC syringeless filters (4 mm × 0.2 μm, Phenomenex, Castel Maggiore, BO, Italy) before injection.

#### HPLC–MS/MS profiling

The phenolic constituents were quantified using a modified version as previously described (Mustafa et al. 2022). Briefly, the dried extracts were sonicated in methanol (5 mg/mL), filtered, and then analyzed using Agilent 1290 Infinity series HPLC–MS/MS system (Agilent Technology, Santa Clara, California, United States), coupled to an electrospray ionization source. The MS/MS parameters of each standard were optimized using flow injection analysis (FIA). The separation of phenolic compounds was accomplished on a Phenomenex Synergi Polar–RP C18 column using water (solvent A) and methanol (solvent B) mixtures with 0.1% formic acid with a flow rate of 0.8 mL/min. Each analyte's most abundant product ions were employed for quantification, while the other ions were used for qualitative analysis. Each compound's unique time window (Δ retention time) was set at 2 min.

### In vivo hepatoprotective activity

#### Animals

Sprague–Dawley healthy adult male rats (180–220 g) and Swiss albino mice from both sexes (25–30 g) were used. All experimental animals were obtained from the breeding unit of the Egyptian Organization of Biological Products and Vaccines (Helwan, Egypt). Animals were housed (3/cage) and maintained at a temperature of 23ºC ± 2ºC with a 12 h light/dark cycle with free access to food pellets and tap water *ad libitum.* The *in vivo* study was implemented following the National Research Council's Guide for the Care and Use of Laboratory Animals and complies with ARRIVE guidelines and NIH animal care protocols. The protocol has been approved by the research ethics, animal care, and use committee of the Faculty of Pharmacy, Helwan University (Regd. No. 01 A2023).

#### Chemicals

PCM was purchased from Alexandria Co. for Pharmaceuticals & Chemicals Industries (Alexandria, Egypt) and silymarin from Sedico Pharmaceutical Co. (6^th^ October City, Giza, Egypt).

#### Acute toxicity study

Healthy adult Swiss albino mice from both sexes (25–30 g) were used for the acute study. Mice were administered orally with different concentrations of the tested extracts reaching up to 5 g/kg. The AMEs were solubilized in distilled water supplemented with 5% Tween 80. Doses were freshly prepared daily during administration to experimental animals following the guidelines on dosage calculation and stock solution preparation in experimental animals’ studies (Earnest and Ajaghaku 2014). General behaviors for mice were observed for 24 h.

#### Experimental design

Fifty-four rats were randomly allocated into nine groups (*n* = 6) as follows:

- Group 1: The control group received the vehicle (distilled water, 1 mL/kg/PO) for one week.
- Group 2: Paracetamol (PCM) group received the vehicle for one week, then received a single dose of PCM (2 g/kg/PO) (Azarmehr et al. 2019) 1 h after the last dose of the vehicle.
- Group 3: The standard group received Silymarin (100 mg/kg/PO) (Hanafy et al. 2016) for one week, then was given a single oral dose of PCM one hour after the last dose.
- Groups 4–9: Treated groups received AME of *A. eggersii, C. macrospermum,* and *J. caffra,* respectively (500 and 1000 mg/kg/PO) for 7 days, then were given a single oral dose of PCM one hour after the last dose. The doses were prepared under the determination of acute toxicity (2.4.3).

Forty-eight hours post administration of PCM, animals were lightly anesthetized using sodium thiopental (40 mg/kg/IP) (El-Kashef and Abdelrahman 2020). Blood samples were aspirated from the retro-orbital plexus by heparinized tubes and then centrifuged for 10 min at 3000 rpm to separate the serum to estimate liver enzymes. Subsequently, rats were sacrificed by cervical dislocation, and livers were immediately removed from the dissected rats, washed with ice-cold saline, dried, and weighed to calculate the liver weight/body weight (LW/BW) ratio. For each rat, a section from the liver tissues was fixed in a formal saline solution (10%) for histopathological analysis. In contrast, another section was homogenized in ice-cold phosphate buffer (pH 7.4, 0.1M), centrifuged (30 min, 3000 rpm and 4 °C) and used for biochemical parameters analysis.

#### Liver function assessments

Liver enzymes such as aspartate aminotransferase (AST), and alanine aminotransferase (ALT), were estimated in serum samples using ELISA Kits (Biomatik, Wilmington, United States, Catalog # EKU02211 and EKE62019, respectively) according to the manufacture protocol.

#### Determination of oxidative stress markers level in liver tissue homogenate

Malondialdehyde (MDA) and reduced glutathione (GSH) were determined by the BioVision ELISA assay kit (Catalog # K739-100, and K464-100, respectively, Milpitas, United States). Superoxide dismutase (SOD) was quantified using a colorimetric kit (Catalog # K335-100, BioVision, Milpitas, USA).

#### Measurement of inflammatory markers in liver tissue homogenate

The levels of tumor necrosis factor-alpha (TNF-α), interleukin-1β (IL-1β), and nuclear factor-kappa B P65 (NF-κB p65) were assayed by ELISA kits (Catalog # 438206, BioLegend, San Diego, United States), (Catalog # SEA563Ra, Cloud-Clone Crop, Texas, United States) and (Catalog # SLD1755Ra, SunLong Biotech, Zhejiang, China), respectively.

#### Determination of apoptotic markers in liver tissue homogenate

BioVision ELISA kit was used for caspase-3 (Catalog # E4592-100, Milpitas, USA). ELISA kits from Cloud-Clone (Houston, USA) were used to determine the levels of BAX (Catalog # SEB343Ra), and Bcl-2 (Catalog # SEA778Ra).

#### Histopathological analysis

Representative liver sections were dissected and fixed in neutral-buffered formalin (10%) for three days, followed by dehydration in gradual ethanol, clearing with xylene, and embedded in paraffin wax. Tissue sections of 4 μm were cut using a rotary microtome, then stained by Harris Hematoxylin and Eosin dye for microscopical histopathological examinations. The procedures of fixation and staining were implemented following the procedure stated earlier (Culling 1974) An experienced pathologist blindly examined samples for lesions or abnormalities using a full HD microscopic imaging system (Leica Microsystems GmbH, Germany). According to El-Nabarawy and co-workers, the tissue lesions were scored as follows: Nil –: no abnormal cellular alterations were demonstrated; + : few lesions were focally demonstrated in 1–3 examined tissue sections; + + : mild lesions were focally demonstrated in 4–6 examined tissue sections. + + + : means moderate lesions were diffusely recorded in 4–6 examined tissue sections. + + + + : severe lesions were diffusely recorded in all the tissue sections examined (El-Nabarawy et al. 2020).

#### Molecular docking

The potential binding mode of the major identified compounds from each secondary metabolites class in three investigated extracts; *Phenolic* acids (Neochlorogenic acid, chlorogenic acid, p-Hydroxybenzoic acid, and Vanillic acid), Flavonols (Rutin, Isoquercitrin, and Hyperoside), Flavanones (Hesperidin) and Flavan-3-ols (Catechin) with caspases-3 and SOD-1 were predicted using molecular docking studies and the PDB ID: 1GFW (Lee et al. 2000) and 5YTO (Manjula et al. 2018). The docking study employed Autodock Vina and OpenBabel tools, while the results visualization employed Discovery Studio. Autodock Vina employs a united atom scoring function and an amber force field. Furthermore, Vina uses a gradient-based local search genetic algorithm and a global optimization algorithm to forecast the binding mode of small molecules to their target. The ligands and proteins were initially prepared and stored in the pdbqt format using OpenBabel tools. Subsequently, the active sites of caspases-3 and SOD-1 were identified by the binding of the co-crystalized ligand (PDB ID: MSI and 946) with the dimensions of 20*20*20 Å in the x, y, and z orientations. Finally, for analysis, the results of the docking scores and binding interactions obtained from the 2D interaction diagrams were conducted.

#### Molecular dynamic simulation method

Molecular dynamic simulations (MDS) were conducted for 100 ns using GROMACS 2.1.1 software (Abraham et al. 2015) on best-scored phenolics. The retrieved docking coordinates of bounded-SOD-1 were used as input structures for molecular dynamics. The receptor and ligand topologies were produced using PDB2 gmx (integrated within GROMACS) and the GlycoBioChem PRODRG2 Server, both utilizing the GROMOS96 force field. Upon reassembling ligands and receptor topologies to construct the system, the standard molecular dynamics protocol of GROMACS was employed for all systems. This encompasses solvation, neutralization, energy reduction utilizing the GROMOS96 43a1 force field, and two phases of equilibration (NVT and NPT). An unrestricted production phase of 100 ns was conducted for the two systems, employing the particle mesh Ewald (PME) method to calculate long-range electrostatic values with a 12 Å cut-off and 12 Å Fourier spacing. Firstly, the simulation trajectories were conducted using GROMACAS to judge the stability of the complexes using RMSD, RMSF, and RG values. The outcomes of the principal component analysis (PCA) were further examined by Gibbs free energy landscape (FEL) calculations. The free energy landscape (FEL) is employed for three-dimensional conformational sampling. The FEL was generated in PyMOL utilizing the geo-measure tool (Kagami et al. 2020). The gmx_sham command is employed to generate 3 FEL energy for calculating the joint probability distribution in three-dimensional space. Dynamic cross-correlation matrix (DCCM) quantifies the correlation coefficient magnitude based on the degree of atomic variation inside the system. Cross-correlation was calculated using the final 10 ns of the molecular dynamics’ simulation trajectory of the protein–ligand complex, employing the Bio3D package in R Studio (Grant et al. 2006).

#### Statistical analysis

Statistical analysis was achieved using GraphPad Prism (version 8, GraphPad Software Inc., San Diego, California, United States). Results were expressed as mean value ± SE. One-way ANOVA and Tukey’s tests were used for multiple comparisons between groups. The value *p* < 0.05 was considered statistically significant.

## Results

### HPLC–MS/MS profiling of three aqueous-alcoholic extracts

The leaves of three palm tree species were exhaustively extracted affording 60.5, 57.2, and 80.4 g dark brown, viscous extracts, respectively. The 2D-chromatographic investigation showed different classes of phenolic metabolites, including, but not limited to, flavonoids and phenolic acids. HPLC-tandem MS (Supplementary figures S1-S3; Table S1) revealed the exclusive qualitative and quantitative identification of eighteen, twenty-five, and twenty-eight metabolites in *A. eggersii,* *C. macrospermum,* and *J. caffra,* respectively (Table 1). The highest phenolic content was detected in *J. caffra* extract (151997.66 μg/Kg) (Table 2). The most abundant class was the phenolic acids that comprised 64793.37, 12637.57, and 10583.55 μg/Kg for *J. caffra*, *C. macrospermum*, and *A. eggersii*, respectively. Other identified phenolic classes are flavanols (73935.79—566.94 μg/Kg), flavan-3-ols (12831.41–84.07 μg/Kg), and flavanone (1575.55—1233.75 μg/Kg) (Table 2). On the other hand, dihydrochalcones were detected in minor amounts of 1.44 and 0.39 μg/Kg in *C. macrospermum* and *J. caffra*, respectively, while they were absent in *A. eggersii.* Anthocyanins and stilbenes were undetectable in the three investigated extracts. Regarding the dominant phenolic constituent in each class, chlorogenic acid pioneered the phenolic acid class in both *C. macrospermum* (7000.057 μg/Kg) and *J. caffra* (47951.511 μg/Kg), while vanillic acid in *A. eggersii* (5900.903 μg/Kg). Rutin is the principal flavonol diglycoside in the three extracts constituting 57970.205, 1427.895, and 365.852 μg/Kg in each of *J. caffra, A. eggersii,* and *C. macrospermum,* respectively. On the otherhand, hyperoside is the principle flavonol monoglycoside detected in *C. macrospermum* (7379.297 μg/Kg) and *J. caffra* (62.764 μg/Kg). Catechin and epicatechin, the hallmarks of the flavan-3-ol class, were denoted in *C. macrospermum* (638.682–101.970 μg/Kg) and *J. caffra* (8731.668–1649.187 μg/Kg) extracts, respectively. Procyanidin B2 was detected in *J. caffra* (2104.496 μg/Kg), while procyanidin A2 was detected in all species. Concurrently, the dihydrochalcones phloridzin and phloretin were detected in *J. caffra* (0.106 and 0.285, respectively), while only phloridzin was noticed in *C. macrospermum* (1.444 mg/k). Lastly, the flavanone glycoside hesperidin was noted in all extracts, being major in *A. eggersii* (1575.550 μg/Kg) and *C. macrospermum* (1225.976 μg/Kg), while naringin was exclusive to *C. macrospermum* and *J. caffra* extracts (306.147 and 7.773 μg/Kg, respectively). Lastly, *trans-*cinnamic acid is the only non-phenolic constituent identified in merely equivalent amounts in all investigated extracts.

**Table 1:** Quantitative profiling (μg/Kg) of phenolic constituents detected in the aqueous methanol extract of *A. eggersii*, *C. macrospermum*, and *J. caffra* leaves by HPLC–MS/MS (RSD% ≤ ± 5.3)

No	Compound/Sample	*A. eggersii*	*C. macrospermum*	*J.caffra*
Phenolic acids				
1	Gallic acid	148.972	76.139	27.793
2	Neochlorogenic acid	6.749	1220.569	14125.853
3	Chlorogenic acid	128.439	7000.057	47951.511
4	p-Hydroxybenzoic acid	2759.858	1946.458	789.670
5	3-Hydroxy benzoic acid	n.d	n.d	n.d
6	Caffeic acid	113.737	128.451	417.772
7	Vanillic acid	5900.903	1689.027	726.923
8	Syringic acid	359.037	184.086	45.670
9	p-Coumaric acid	999.887	174.679	288.708
10	Ferulic acid	67.156	9.720	19.219
11	3,5-Dicaffeoylquinic acid	0.834	5.946	344.577
12	Ellagic acid	97.975	202.436	55.674
Flavonoids				
(A) Anthocyanins				
13	Delphinidin 3,5 diglucoside	n.d	n.d	n.d
14	Delphinidin3-galactoside	n.d	n.d	n.d
15	Cyanidin-3-glucoside	n.d	n.d	n.d
16	Petunidin-3-glucoside	n.d	n.d	n.d
17	Pelargonidin-3-rutinoside	n.d	n.d	n.d
18	Pelargonidin-3-glucoside	n.d	n.d	n.d
19	Malvidin-3-galactoside	n.d	n.d	n.d
(B) Flavonols				
20	Rutin	1427.895	365.852	57970.205
21	Isoquercitrin	0.000	55.229	6756.219
22	Quercitrin	4.693	1.643	14.080
23	Myricetin	n.d	n.d	n.d
24	Kaempferol-3-glucoside	n.d	31.903	1363.319
25	Quercetin	8.654	29.645	303.996
26	Isorhamnetin	3.461	19.903	22.318
27	Hyperoside	n.d	62.764	7379.297
28	Kaempferol	n.d	n.d	126.355
(C) Flavan-3-ols				
29	Catechin	n.d	638.682	8731.668
30	Epicatechin	n.d	101.970	1649.187
31	Procyanidin B2	n.d	n.d	2104.496
32	Procyanidin A2	84.073	102.630	346.055
(D) Dihydrochalcones				
33	Phloridzin	n.d	1.444	0.106
34	Phloretin	n.d	n.d	0.285
(E) Flavanones				
35	Hesperidin	1575.550	1225.976	100.725
36	Naringin	n.d	7.773	306.147
Stilbenes				
37	Resveratrol	n.d	n.d	n.d
Non-Phenolic acids				
38	Trans-cinnamic acid	24.193	28.715	29.770

n.d., not detected

**Table 2:** Quantitative estimation (μg/Kg) of various phenolic classes identified in the aqueous methanol extract of *A. eggersii, C. macrospermum*, and *J. caffra* leaves using HPLC–MS/MS

Content	*A. eggersii*	*C. macrospermum*	*J. caffra*
Anthocyanins	-	-	-
Flavonols	1444.70	566.94	73935.79
Flavan-3-ols	84.07	843.28	12831.41
Dihydro chalcones	0.00	1.44	0.39
Flavanone	1575.55	1233.75	406.87
Phenolic acids	10583.55	12637.57	64793.37
Total phenolic content	13712.72	15311.98	151997.66

### Acute toxicity study

Oral administration of AMEs of *A. eggersii*, *C. macrospermum,* and *J. caffra* up to 5 g/kg demonstrated neither obvious toxic signs nor death. Since the general behavior of the treated mice did not display any related changes, the tested AMEs are considered safe until the maximum tested dose (5 g/kg). So according to Semler (1992), the extract is considered safe if there is no toxic effect on mice at a dose of up to 5 g/kg (Semler 1992). Consequently, two serial doses (500 and 1000 mg/kg) were selected for the hepatoprotective study based on reported recommendations for testing a phenolic-rich AME (Mady et al. 2022).

### Effect of AME of A. eggersii, C. macrospermum, and J. caffra on LW/BW ratio in PCM-intoxicated rats

Table (3) shows that treatment with PCM significantly increased liver weight and the LW/BW ratio by 1.8 folds and 1.7 folds, respectively, compared to the control rats. Silymarin and AMEs of *A. eggersii, C. macrospermum,* and *J. caffra* at doses 500 and 1000 mg/kg significantly decreased the liver weight by 15.7%, 25.7%, 31.7%, 25.5%, 27.5%, 29.1%, and 29.1%, respectively, and the LW/BW ratio by 16.7%, 21.8%,29.7%, 19.1%, 21.5%, 24.3%, and 29.7%, respectively, compared to the PCM-treated group. The highest protective effect was observed for AMEs of *A. eggersii* and *J. caffra* in the dose of 1000 mg/kg, as they showed a significantly (*P* < 0.05) lower LW/BW ratio than the standard silymarin group, unlike the other treated groups.

**Table 3:** Effect of the aqueous methanol extract of *A. eggersii*, *C. macrospermum*, and *J. caffra* on liver weight/body weight (LW/BW) ratio in PCM-intoxicated rats

Groups	Dose mg/kg	Liver weight (g)	Body weight (g)	LW/BW (%)
Control		4.67 ± 0.23	178.80 ± 4.09	2.61 ± 0.09
PCM	2 g/kg	8.30 ± 0.18	187.80 ± 8.38^a^	4.45 ± 0.017^a^
Silymarin	100	7.00 ± 0.11	189.00 ± 5.25^a,b^	3.71 ± 0.07 ^a,b^
*A. eggersii*	500	6.17 ± 0.19	177.20 ± 3.44^a,b,c^	3.48 ± 0.10^a,b,c^
	1000	5.67 ± 0.10	184.00 ± 10.55^a,b,c^	3.13 ± 0.18^a,b,c^
*C.macrospermum*	500	6.12 ± 0.17	171.70 ± 3.05^a,b,c^	3.60 ± 0.08^a,b^
	1000	6.02 ± 0.15	172.00 ± 2.16^a,b,c^	3.49 ± 0.06^a,b^
*J. caffra*	500	5.88 ± 0.12	175.00 ± 5.59^a,b,c^	3.37 ± 0.09^a,b^
	1000	5.88 ± 0.19	187.80 ± 4.83^a,b,c^	3.13 ± 0.07^a,b,c^

Data have shown as mean ± SE, *n* = 6 rats, a: significant (*p* < 0.05) from the control group, b: significant (*p* < 0.05) from the PCM group, and c: significant (*p* < 0.05) from the silymarin group

### Effect of AME of A. eggersii, C. macrospermum, and J. caffra on liver enzymes in PCM-intoxicated rats

**Fig. 1:**
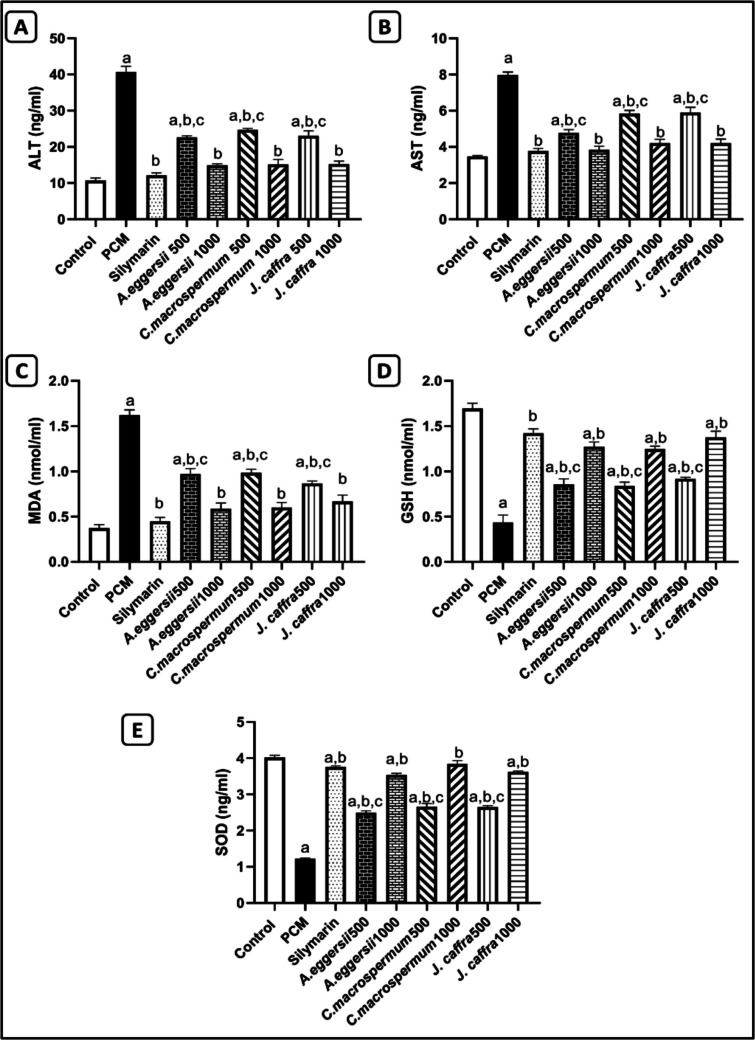
Effect of the aqueous methanol extract of *A. eggersii*, *C. macrospermum,* and *J. caffra* leaves on the liver enzymes and the oxidative stress markers in PCM-intoxicated rats. (**A**): Alanine aminotransferase (ALT), (**B**): Aspartate aminotransferase (AST), (**C**): Malondialdehyde (MDA), (**D**): Reduced glutathione (GSH), (**E**): Superoxide dismutase (SOD). Results were presented as: Mean ± SE, (*n* = 6 rats). a: significant (*p* < 0.05) from the control group, b: significant (*p* < 0.05) from the PCM group, and c: significant (*p* < 0.05) from the silymarin group

As demonstrated in Fig. (1A and B), PCM causes hepatocellular damage evidenced by the significant increase in the liver enzymes ALT and AST by 3.8 folds and 2.3 folds, respectively, compared to the control rats. The oral pretreatment with silymarin and AMEs of *A. eggersii*, *C. macrospermum,* and *J. caffra* at the intended tested doses significantly (*P* < 0.05) reduced the serum levels of ALT by 70.2%, 44.4%, 63.4%, 39.3%, 62.8%, 43.3%, 62.5%, respectively, and AST by 52.7%, 40.2%, 51.9%, 26.8%, 47.3%, 25.9%, and 47.3%, respectively, in comparison to the PCM group. Interestingly, the maximum tested dose (1000 mg/kg/po) of the three AMEs reduced the serum levels of the indicated parameters to levels that are non-significant from the silymarin-standard group. Ultimately, the best protective effect on liver enzyme was observed in rats administered the AME of *A. eggersii* at a dose of 1000 mg/kg/po.

### Effect of AME of A. eggersii, C. macrospermum, and J. caffra on the oxidative stress markers in PCM-intoxicated rats

Compared to the control group, PCM causes a significant increase in MDA by 4.3 folds and a significant decrease in GSH and SOD by 74.2% and 69.6%, respectively (Fig. 1C, D, and E). Treatment with silymarin and the AMEs of the three species at the two tested doses significantly (*P* < 0.05) decreased MDA by 72.4% 40%, 63.8%, 39.3%, 63%, 46.5%, and 58.8%, respectively, and significantly (*P* < 0.05) increased GSH by 3.3 folds, 2.0 folds, 2.9 folds, 1.9 folds, 2.9 folds, 2.1 folds, 3.2 folds, respectively, and SOD by 3.1 folds, 2.0 folds, 2.9 folds, 2.2 folds, 3.1 folds, 2.2 folds, and 3.0 folds, respectively, compared to the PCM-treated rats. Notably, AMEs of the three species at the maximum tested dose (1000 mg/kg/po) showed a non-significant difference compared to the silymarin treatment.

### Effect of AME of A. eggersii, C. macrospermum, and J. caffra on the inflammatory markers in PCM-intoxicated rats

**Fig. 2:**
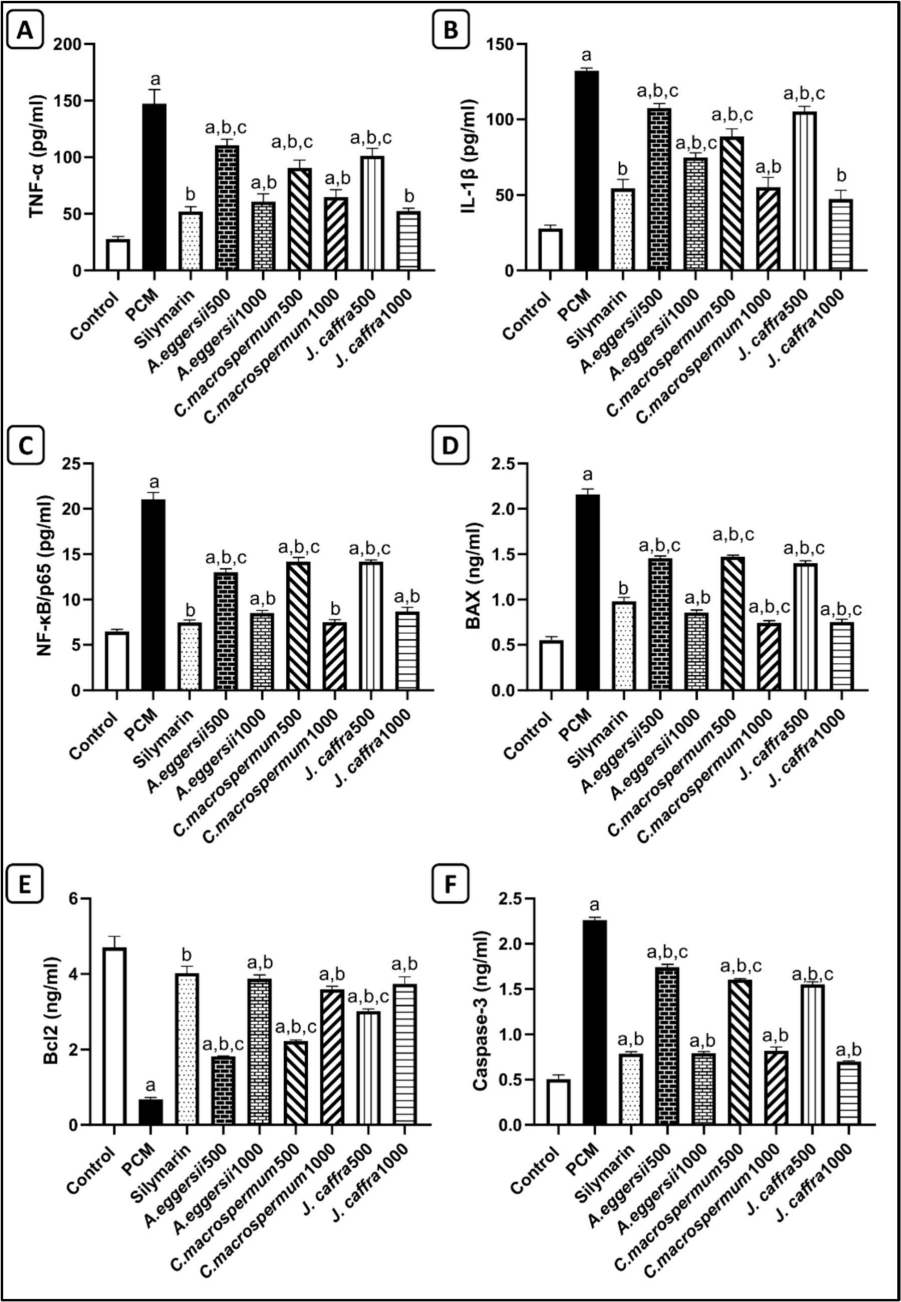
Effect of the aqueous methanol extract of *A. eggersii*, *C. macrospermum,* and *J. caffra* leaves on the inflammatory and apoptotic markers in PCM-intoxicated rats. (**A**): Tumor necrosis factor-alpha (TNF-α), (**B**): Interleukin-1beta (IL-1β), (**C**): Phosphorylated nuclear factor-kabba B (NF-κB/p65), (**D**): BAX, (**E**): Bcl2, (**F**): Caspase-3. Results were presented as: Mean ± SE, (*n* = 6 rats). a: significant (*p* < 0.05) from the control group, b: significant (*p* < 0.05) from the PCM group, and c: significant (*p* < 0.05) from the silymarin group

The results demonstrated that the oral administration of PCM at the intended dose (2 g/kg) significantly increased the phosphorylation of NF-κB and the levels of TNF-*α* and IL-1β by 3.3 folds, 5.3 folds, and 4.8 folds, respectively (Fig. 2A, B, and C). At the same time, the silymarin-supplemented group showed a significant decrease in the levels of p-NF-κB and both cytokines compared to the PCM group by 64.5%, 64.6%, and 58.9%, respectively. Notably, animal groups pre-treated with the AMEs showed a significant (P < 0.05) decrease in the levels of phosphorylated NF-κB by 38.3%, 59.6%, 32.8%, 64.5%, 32.7%, and 58.8%, respectively, and TNF-α levels by 24.9%, 58.6%, 38.5%, 56%, 31.3%, and 64.4%, respectively, and IL-1β levels by 18.7%, 43.4%, 33%, 58.3%, 20.4%, and 64.2%, respectively, compared to PCM-treated group. The effect of 1000 mg/kg of the extracts on TNF-α and NF-κB was nonsignificant from the silymarin group. Additionally, the dose of 1000 mg/kg of *C. macrospermum*, and *J. caffra* on IL-1β is nonsignificant from the silymarin-treated group.

### Effect of AME of A. eggersii, C. macrospermum, and J. caffra on the apoptotic markers in PCM-intoxicated rats

The results showed that PCM caused a significant increase in BAX and caspase-3 by 3.9 folds and 4.5 folds, respectively, and a significant decrease in Bcl2 by 85.7%, compared to the control group. Meanwhile, groups treated with silymarin and the AMEs showed a significant (P < 0.05) decrease in BAX by 54.7%, 32.6%, 60.4%, 31.8%, 65.6%, 35.2%, and 65.2%, respectively, and caspase-3 by 65.1%, 23%, 64.9%, 36.4%, 63.7%, 38.7%, and 69%, respectively, and an increase in Bcl2 levels by 6 folds, 2.7 folds, 5.7 folds, 3.3 folds, 5.3 folds, 4.5 folds, and 5.5 folds, respectively, compared to the PCM group. The effect of 1000 mg/kg of the extracts on Bcl2 and caspase-3 was nonsignificant from the silymarin group. Meanwhile, the dose of 1000 mg/kg of *C. macrospermum,* and *J. caffra* on BAX is significantly lower than the silymarin-treated group (Fig. 2D, E, and F).

### Effect of AME of A. eggersii, C. macrospermum, and J. caffra on histopathological changes in PCM-intoxicated rats

**Fig. 3:**
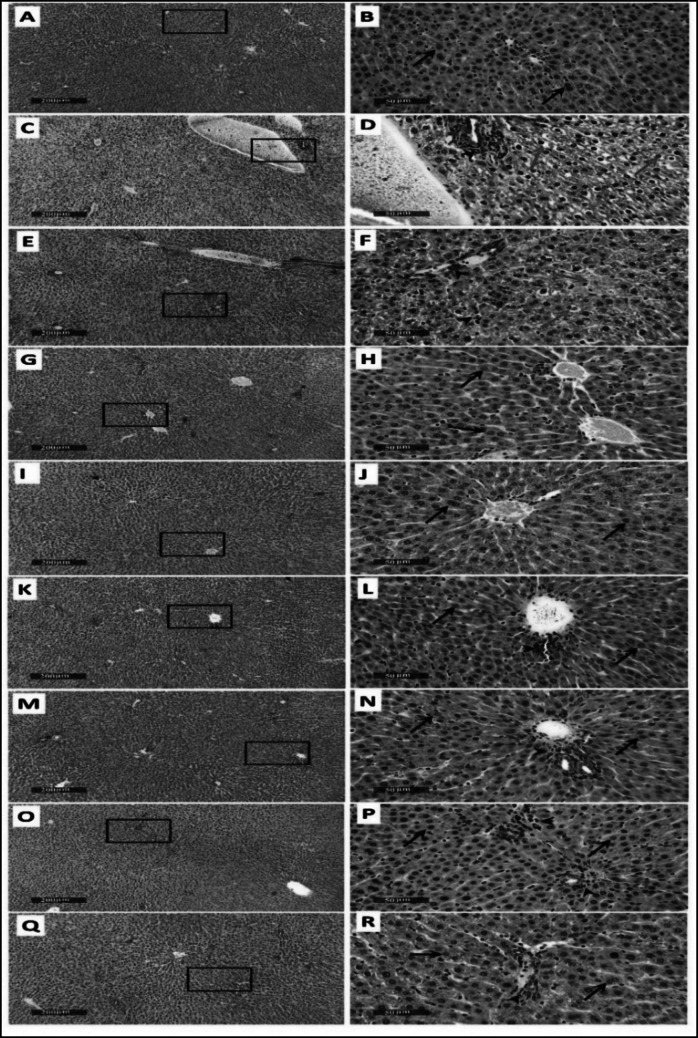
Effect of the aqueous methanol extract of *A. eggersii*, *C. macrospermum,* and *J. caffra* leaves on histopathological changes in PCM-intoxicated rats. **A**&**B**: Control, **C**&**D**: PCM, E&F: silymarin, **G**&**H**: *A. eggersii* 500 mg/kg, I&J: *A. eggersii* 1000 mg/kg, K&L: *C. macrospermum* 500 mg/kg, M&N: *C. macrospermum* 1000 mg/kg, **O**&**P**: *J. caffra* 500 mg/kg and **Q**&**R**: *J. caffra* 1000 mg/kg. H&E stain 100X & 400X. The black arrow = intact hepatocytes, the red arrow = damaged hepatocytes, and Arrowhead = inflammatory cell infiltrates

Microscopical examination of liver sections from the control group revealed normal liver architecture **(**Fig. 3A and B). However, liver sections from the PCM-treated group showed diffuse vacuolar cytoplasmic degeneration that affects the hepatic lobular zones, significant dilation of hepatic vasculatures, and mildly focal mononuclear inflammatory cell infiltrates (Fig. 3C and D). Liver sections examined from the silymarin-treated rats showed mild persistent records of degenerated hepatocytes alternated with higher records of apparent intact cells with mild focal inflammatory cell infiltrates (Fig. 3E and F). Conversely, obvious hepatoprotective efficacy was detected in liver sections obtained from experimental rats pretreated with *A. eggersii* at 500 mg/kg/po (Fig. 3G and H) and 1000 mg/kg/po (Fig. 3I and J). That is inferred from the reduction or absence of inflammatory cells, vascular congestion, cellular degeneration, necrosis, and vacuoles in the examined liver sections. Moreover, mild focal periportal inflammatory cells with intact hepatocytes and vasculatures were observed in some liver sections at 500 mg/kg and 1000 mg/kg treatment doses of *C. macrospermum* (Fig. 3K-N), and *J. caffra* (Fig. 3O-R). The lesion score of the examined tissues is demonstrated in Table (4)

**Table 4:** The scores of histological alterations of liver tissue in the tested groups

	Control	PCM	Silymarin	*A. eggersii*		*C. macrospermum*		*J. caffra*	
Dose (mg/kg)		2 g/Kg	100	500	1000	500	1000	500	1000
Degenerative/necrotic changes	-	+ + + +	+ +	-	-	-	-	-	-
Vascular dilation	-	+ + +	+	-	-	-	-	-	-
Inflammatory cells infiltrate	-	+ +	+ +	-	-	+ +	+	+ +	+

### Evaluating the binding affinity of the major identified polyphenols through molecular docking

Re-docking the co-crystallized ligand (PDB ID: 964) into the binding site of caspase-3 (PDB ID: 1GFW) confirmed the validity of the molecular docking technique. S score of −6.19 kcal/mol and RMSD values of 1.04 were recovered from the PDB-generated original poses. We recognized that all the docked polyphenols occupied the binding pocket and superimposed the co-crystallized ligand with RMSD values and binding energy scores as follows; Neochlorogenic acid (1.54 Å, −5.7899 kcal/mol), chlorogenic acid (0.94 Å, −6.0998 kcal/mol), *p*-hydroxybenzoic acid (0.28 Å, −4.3556 kcal/mol) and vanillic acid (1.12Å, −3.9653 kcal/mol), rutin (1.27Å, −8.0671 kcal/mol), isoquercitrin (1.34 Å, −6.3170 kcal/mol) and hyperoside (1.32 Å, −6.5343 kcal/mol), hesperidin (0.80 Å, −7.1735 kcal/mol) and catechin (1.015 Å, −5.04 kcal/mol) (Table 5).

**Table 5:** Results of molecular docking of the Crystallized Ligand and most identified compounds in conjunction with SOD1 (PDB ID: 5YTO) and caspase-3 (PDB ID: 1GFW)

	PDB ID: 5YTO			PDB ID: 1GFW		
	RMSD (kcal/mol)	Score (Å)	Interacting amino acids	RMSD (kcal/mol)	Score (Å)	Interacting amino acids
Crystallized Ligand	−6.90	1.01	**H-bond** Ser98, Ile99 **π anion** Glu21, Glu100 **π-π staked** Trp32	−6.1982	1.04	**H-bond** GlyA122, HisA121, ArgB207 **π-π T-Shaped** TyrB204 **π-alkyl** CysA163, TrpB206
Phenolic acids						
Chlorogenic acid	−6.67	1.34	**H-bond** Thr2, Glu21, Val3, Glu100 **Amide π staked** Ile99 **π-Donor H-bond** Trp32	−5.7899	1.54	**H-bond** GlyA122, HisA121 ArgB207, SerB205 **π-donor H-bond** PheB256, TrpB206 **π-π T-Shaped** TyrB204 **π-δ** MetA61 **π-alkyl** CysA163
Neochlorogenic acid	−6.44	1.00	**H-bond** Pro28, Glu21, Lys30, Glu100 **π-Donor H-bond** Gln22, Trp32 **π-Alkyl** Lys23	−6.0998	0.94	**H-bond** GluA123, HisA121 ArgB207, SerB205 **π-donor H-bond** TrpB206 **π-δ** MetA61 **π-alkyl** TyrB204
Vanillic acid	−4.45	1.11	**H-bond** Thr2, Glu100 **Carbon H bond** Ile99 **Attractive charge** Lys3 **π-Alkyl** Lys30	−4.3556	0.28	**Attractive charge** ArgA64, HisA121 ArgB207 **π-donor H-bond** TrpB206 **π-π staked** TryB204 **π-alkyl** CysA163, PheB256
4-Hydroxy benzoic acid	−4.42	1.64	**H-bond** Thr2, Glu21, Glu100 **π-Alkyl** Lys3	−3.9653	1.12	**H-bond** HisA121 **Attractive charge** ArgA64, HisA121 ArgB207 **π-π staked** TryB204, TrpB206
Flavonols						
Rutin	−8.24	1.71	**H-bond** Thr2, Pro28, Ser98, Ile99, Glu100 **π anion** Glu21, Glu100 **π-Donor H-bond** Trp32 **π-Alkyl** Lys23, Lys30, Trp32	−8.0671	1.27	**H-bond** GlyA122, HisA121, ArgB207, SerB205, Asn208 **π-donor H-bond** TrpB206 **π-π T-Shaped** TyrB204 **π-alkyl** CysA163
Hyperoside	−7.14	1.0	**H-bond** Pro28, Ser98, Glu100 **π anion** Glu21, Glu100 **π-Donor H-bond** Trp32 **π-Alkyl** Lys23, Lys30	−6.3170	1.34	**H-bond** GlyA122, HisA121 TyrB204, ArgB207 **π-δ** MetA61 **π-π T-Shaped** TyrB204 **π-alkyl** CysA163
Isoquercitrin	−7.77	1.44	**H-bond** Thr2, Glu21, Pro28, Val31, Ile99, Glu100 **π anion** Glu21 **π-Donor H-bond** Trp32 **π-Alkyl** Lys23, Lys30	−6.5343	1.32	**H-bond** GlyA122, HisA121, ArgB207, SerB205, TryB204 **π-donor H-bond** TrpB206 **π-δ** MetA61 **π-π T-Shaped** TyrB204 **π-π staked** TryB204, PheB256 **π-alkyl** CysA163
Flavanones						
Hesperidin	−8.36	0.91	**H-bond** Gln22, Ser98, Glu100, Glu132 **π anion** Glu21, Glu100 **π-Alkyl** Trp32, Pro74, Lys75	−7.1735	0.80	**H-bond** HisA121, CysA163, GlyA165, TyrB204,ArgB207 **π-donor H-bond** AsnB208 **π-alkyl** TrpB206, MetA61
Flavan-3-ols						
Catechin	−5.88	0.77	**H-bond** Glu21, Pro28, Lys30, Ile99, Glu100 **π anion** Glu21 **Carbon H-bond** Ser98 **π-Alkyl** Lys23, Lys30	−5.0457	1.01	**H-bond** HisA121, ArgB207 **π-π T-Shaped** TyrB204 **π-alkyl** CysA163

Regarding the SOD1 (PDB ID: 5YTO) *in silico* docking study, the co-crystallized ligand (PDB ID: MSI) along with the major identified natural compounds were docked into SOD1 binding sites (PDB code: 5YTO) and the results (Table 5) showed that flavonol rutin, isoquercitrin, and hyperoside, and the flavanones hesperidin achieved excellent fitting with a highly efficient binding score of −8.24, −7.14, −7.77 and −8.36 kcal/mol; respectively compared to co-crystallized ligand (PDB ID: MSI) (−6.9 kcal/mol). In comparison, the phenolic acids (neochlorogenic, chlorogenic, *p*-hydroxybenzoic, and vanillic), and flavan-3-ols (catechin) showed an acceptable binding affinity with docking scores −6.67, −6.44, −4.45, −4.42 and −5.88 kcal/mol, respectively.

## Discussion

The three palm tree species (Family Arecaceae) were selected based on two criteria. Firstly, as a research team we are interested in discovering hepatoprotective alternative scaffolds inspired by phenolics-rich natural products (Elsayed et al. 2022). Secondly, there are several reports about Arecaceae family species possessing promising hepatoprotective potential either as traditional practice (Gruca et al. 2015) or scientifically proven (Pithayanukul et al. 2009; Sasidharan, et al. 2009; EI Arem et al. 2014; El-Ghonemy et al. 2019; Fatani et al. 2022; Saafi et al. 2010; Nageh et al. 2022). Accordingly, we preliminarily screened the phytochemical composition (2D-PC) of several unexplored Arecaceae species cultivated in Egypt, and we identified three species: *A. eggersii*, *C. macrospermum,* and *J. caffra* as the focus of the study from both pharmacological and phytochemical aspects. Regarding the potential of the three tested extracts in the PCM-induced liver injury animal model, the model is a well-documented, straightforward, and easy-to-handle protocol commonly used to assess the intrinsic DILI. Hence, we evaluated the hepatoprotective activity of the tested extracts using a PCM-induced liver injury in experimental rats. Herein, the oral administration of PCM at a toxic dose of 2 g/kg caused liver toxicity. The observed significant changes in LW linked with liver enlargement are due to the progression of hepatic lesions and toxicity with the subsequent accumulation of extracellular matrix protein and collagen in liver tissue (Saad et al. 2013; Mahmood et al. 2014). The induced hepatotoxicity seems to be contingent, at least in part, on the formation of free radicals and oxidative processes from the highly reactive NAPQI (PCM metabolite). Free radicals affect the cellular membrane, induce lipid peroxidation (Chen et al. 2009), and trigger the escaping of cellular enzymes into the bloodstream (Yahya et al. 2013). Interestingly, several studies have supported the claim that the hepatoprotective effect and radical scavenging activity of natural products, especially polyphenols, synergistically inhibit the progression of hepatocellular injury (Mahmood et al. 2014; Chen et al. 2009; Yahya et al. 2013). This makes polyphenols the center of attention for the treatment protocols for several liver problems. Witnessed by silymarin, which is one of the most researched plant extracts that has been engaged in the protective treatment of liver disorders and adopted as a positive control drug in many studies (Flora et al. 1998). In our study, the animal groups pretreated with the AME of *A. eggersii, C. macrospermum*, and *J. caffra* showed promising hepatoprotective effects. The AME at different doses successfully reduced the liver weight, LW/BW ratios, liver enzymes, inflammatory markers, and apoptotic markers while increasing the GSH and SOD in a dose-dependent manner. Our data coincides with the renowned hepatoprotective reports on family Arecaceae species. For instance, Abdelaziz and Ali reported the hepatoprotective potential of *Phoenix dactylifera* seeds in CCl_4_-induced hepatotoxicity animal protocol by normalizing serum levels of AST, ALT and increasing SOD and GST (Abdelaziz and Ali 2014). Additionally, Soundararajan and co-authors have demonstrated the *in vivo* hepatoprotective effect of *Elaeis guineensis* in paracetamol-induced liver toxicity which showed a decrease in AST, ALT, and bilirubin (Abdelaziz and Ali 2014; Soundararajan et al. 2012).

Meanwhile, the tandem HPLC–MS analysis showed their profusion in various polyphenol classes, such as flavonoids and phenolic acids. Polyphenols have shown intriguing antioxidant and anti-inflammatory activities, *in vitro *and* in vivo*, through their potential free radical scavenging and inhibition of inflammatory mediators (Zhang and Tsao 2016). Their activity is interrelated to specific structural features comprising the presence of an *ortho-*catechol system or 5-OH adjacent to a carbonyl group in flavonoids and a free or substituted phenolic group(s) in the phenolic acid (Rice-Evans et al. 1996; Sekher Pannala et al. 2001). Phenolic groups have a strong reducing capacity, so they can easily donate hydrogen, scavenge the free radicals, neutralize them, and prevent lipid peroxidation (Wolf et al. 2005). This protective effect mechanism helps to maintain cell membrane integrity, preserve the store of GSH, and activate various anti-inflammatory pathways (Shimoda et al. 2008). Mechanisms of hepatoprotection may include activation of liver regeneration by enhancing the protein and glycoprotein synthesis or accelerated detoxification and excretion (Kumar et al. 2004). Interestingly, these pharmacophore features are established in most of the identified phenolic compounds in the three extracts, with several reports in the literature highlighting their antioxidant, anti-inflammatory, and hepatoprotective actions. For instance, rutin, a diglycoside flavonoid that is detected in the three investigated species in variable amounts, has a noteworthy role in opposing cellular oxidative stress, regulating lipid metabolism, possessing an anti-inflammatory, anti-apoptotic effect, and restoring liver functions (El-Maadawy, et al. 2021; Gęgotek et al. 2019; Rahmani et al. 2023).

One more example is hesperidin, an identified flavanone in the AME of both *A. eggersii* and *C. macrospermum*. Several studies have documented that hesperidin possesses antioxidants, anti-inflammatory, and anti-apoptotic properties (Tirkey et al. 2005). It reduced the serum levels of AST, ALT, NF-_k_B, TNF-α, MDA, Bax, and Caspase3 content, while increasing the SOD, GSH, IL-10, and Bcl2 (Chen et al. 2013; Rabee and Hassen 2018). On the other side, epicatechin and catechin are two isomeric flavanols that are abundant in the AME of *J. caffra* and, to a lesser extent, in *C. macrospermum.* Alkinani and his research team (Alkinani et al. 2021) have reported the hepatoprotective effects of (-)-epicatechin in a CCl4-induced toxicity model. The study showed that epicatechin positively modulates liver functions by decreasing the serum levels of ALT and AST and increasing the levels of TP and serum albumin. Also, epicatechin combined with silymarin reduced MDA levels, while upregulating the iNOS and cytochrome P450 (CYP450) levels. Moreover, they improved the histopathological alterations caused by the administration of the xenobiotic, CCl_4_. They reduce oxidative stress, suppress inflammatory cell infiltration, increase the regenerative capacity of damaged tissues, and reduce liver apoptosis. Bernatoniene and Kopustinskiene (2018) have summarized, in their collective review, the biochemical and antioxidant properties of catechins which exert their antioxidant effect either directly by scavenging reactive oxygen species (ROS) and metal ions chelation or indirectly by inducing antioxidant enzymes, inhibiting pro-oxidant enzymes, and producing phase II detoxification enzymes and antioxidant enzymes. Skimming the HPLC–MS/MS, we acknowledged the presence of major phenolic acids such as vanillic, and chlorogenic that are accredited in the literature for their antioxidant, anti-inflammatory, and hepatoprotective effects. Interestingly, Khedr et al. (2014) have reported the hepatoprotective effect of vanillic acid in a thioacetamide-induced hepatotoxicity model. In this investigation, vanillic acid significantly reduced the serum AST and ALT enzymes, restored the abnormal levels of MDA, and declined the levels of inflammatory markers with consistent histopathological observation. On the other side, Cheng et al. (2023) documented the modulation of chlorogenic acid for oxidative stress and inflammation. Chlorogenic acid supplementation reduced the serum levels of liver enzymes, MDA content, and pro-inflammatory cytokine synthesis, it prevented histopathological changes in the liver and increased GSH levels, catalase activity, and IL10 levels. Additionally, Moslehi et al. (2023) documented the role of chlorogenic acid in attenuating liver apoptosis and inflammation in endoplasmic reticulum stress-induced mice model via inhibiting the inflammatory markers NF*-κ*B*,* and TNF*-α,* in addition to the apoptotic markers caspase 3 and Bax. Interestingly, most of the forementioned phenolics have been previously detected and quantified in other Arecaceae species.

Ultimately, although the identified bioactive flavonoids and phenolic acids were quantified in the three extracts with variable concentrations, we could hypothesize the possibility of synergistic influence between the phenolic components of the total extract, although further investigation is required.

**Fig. 4:**
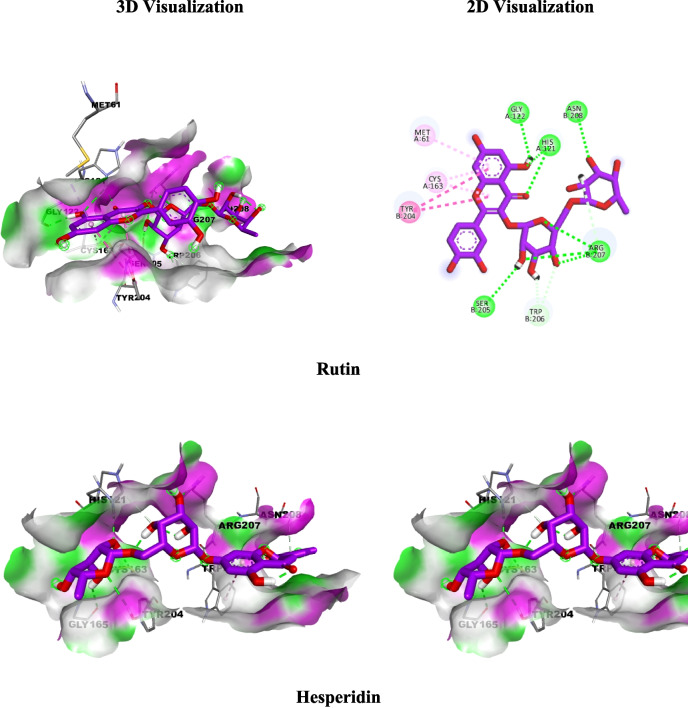
3D (Left) and 2D (Right) structure interaction poses of caspase 3 (PDB ID: 1GFW) with the Rutin and Hesperidin as major identified flavonoids with the best binding energy

Molecular docking was performed to analyze the possible interactions of selected, major identified polyphenols with caspase 3 and SOD-1. The caspase family is a group of aspartate cysteine proteases that play critical roles in apoptosis and numerous physiological and pathological processes (Kemnitzer et al. 2004). The active site as well as the S2 and S3-pockets, are visible in the caspase-3 crystal structure in complex with the inhibitor, Isatin sulfonamide derivative. The co-crystallization of the inhibitor into the active site of caspase-3 demonstrates that the methylamine on the isatin moiety interacts with the MetA61 amino acid residue in the target site pocket (Fig. 4S). The interaction between CysA163 and GlyA122 is facilitated by the hydrogen bonds formed by the carbonyl groups. Caspase-3's S2 and S3 compartments proved suitable for Isatin’s pyrrolidinyl and phenoxy scaffolds with subsequent hydrophobic contacts with the Ph256 residues. Concerning our major, identified polyphenols in the AME, the flavonol pharmacophore of Rutin, Isoquercitrin, and Hyperoside generate two main hydrogen bonding interactions with HisA121 and GlyA122. Additionally, they exhibit various *pi*-stacking, like T-shaped π–π interaction with TyrB204, π-δ with MetA61, and π-alkyl with CysA163 (Fig. 4 and 4S). Meanwhile, the primary sugar moiety for the three flavonols forms an H-bonding with ArgB207; Rutin, and Isoquercitrin promote an H-bond with SerB205. The secondary sugar moiety in Rutin extends to generate extra H-bond with Asn208 which might be the rationale for the best binding energy at the target pocket. Concurrently, the substituted aromatic ring of Isoquercitrin plunges into the hydrophobic pocket PheB256. On the other hand, Hesperidin displays the best binding energy at the target pocket as the disaccharide moiety generates five hydrogen bonds at the active site. In addition, Hesperidin aglycon’s scaffold extended to the S2 pocket (neighboring the hydrophobic residue TyrB204), while the phenolic OH extended to form hydrogen bonding with Asn208 (Fig. 4). Concerning polyphenolic compounds other than flavonoids, Neochlorogenic, and Chlorogenic acids fulfill all the major interactions. The Dihydrochalcone possesses a carbonyl scaffold and displays critical function in potentially promoting its location at the binding site. Quinic acid expands to create an extra H-bonding with SerB205 (Fig. 4S). Catechin, *p*-Hydroxybenzoic acid, and Vanillic acid fit in the binding pocket fulfilling some of the major interactions, however the compound's size cannot expand to occupy S2 and S3 pockets which explains its low binding energy (Fig. 4S).

**Fig. 5:**
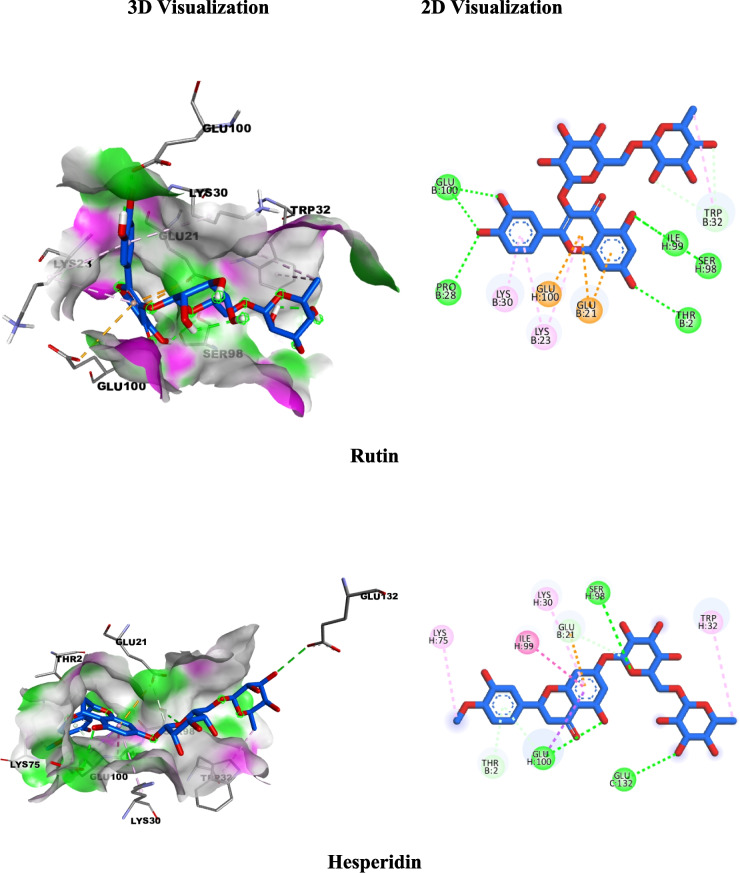
3D (Left) and 2D (Right) structure interaction poses of SOD1 (PDB ID: 5YTO) with Rutin and Hesperidin identified in the AME of the three species and display the best binding energy

The homodimer metalloenzyme superoxide dismutase 1 (SOD1) is a crucial component of the physical endogenous antioxidant defense mechanism and facilitates the transformation of superoxide anion into hydrogen peroxide. Among the emerging techniques for the invention of a therapeutic drug for mutations in SOD1-related disorders is the stabilization of the SOD1 dimer interface. SOD1 interacts with a class of chemicals that includes the catecholamine neurotransmitters (dopamine and epinephrine) *via* a ligand-binding site on the surface of the –barrel (Manjula et al. 2018; Ray et al. 2005). Herein, we utilized the X-ray crystal structure of SOD1 bounded to a naphthalene-catechol-linked molecule (PDB ID: 5YTO) to conduct molecular docking simulations with the major identified compounds. Catechol was identified in a hydrophilic pocket generated by SOD1 loop II lined by Glu21, Pro28, Gln22 Ser98, and Glu100 which interact with a catechol OH. The carbonyl group of the Glu21 side chain may also interact with π-anion assembling, and the naphthalene group π-π stacking with Trp32. Concerning the major identified polyphenols, they generally occupy the hydrophilic pocket lined with Thr2, Lys30, Lys23, Glu21, Pro28, and Gln22 residues, overlay the crystalized molecule at the dimer phase, display the major H-bonding, and extend to interact with Trp32 as shown in supplementary Fig. 5S. The aglycon moiety of Rutin, Isoquercitrin, and Hyperoside impeded in the hydrophilic pocket and generated H-bonding with Thr2, Pro28, Ser98, Ile99, and Glu100. Moreover, the disaccharide moiety of rutin extended to interact with the hydrophobic moiety of trp32 which is responsible for the best binding energy. Hesperidin displays the same binding pattern as Rutin and possesses a high binding score (Fig. 5). The phenolic acids (Chlorogenic and Neochlorogenic) and Flavan-3-ols (Catechin) generate the H-bonding in the hydrophilic pocket and extend to approach the Trp32 with moderate binding energy. Finally, the vanillic and 4-hydroxybenzoic acids failed to interact with Trp32 due to their small size even though producing the H-bond at the hydrophilic pocket (Supplementary Fig. 5S). These findings suggest that the percentage of dominant phenolic compounds with potential docking scores authenticate caspases 3 and SOD-1 as possible targets for the three AMEs.

**Fig. 6:**
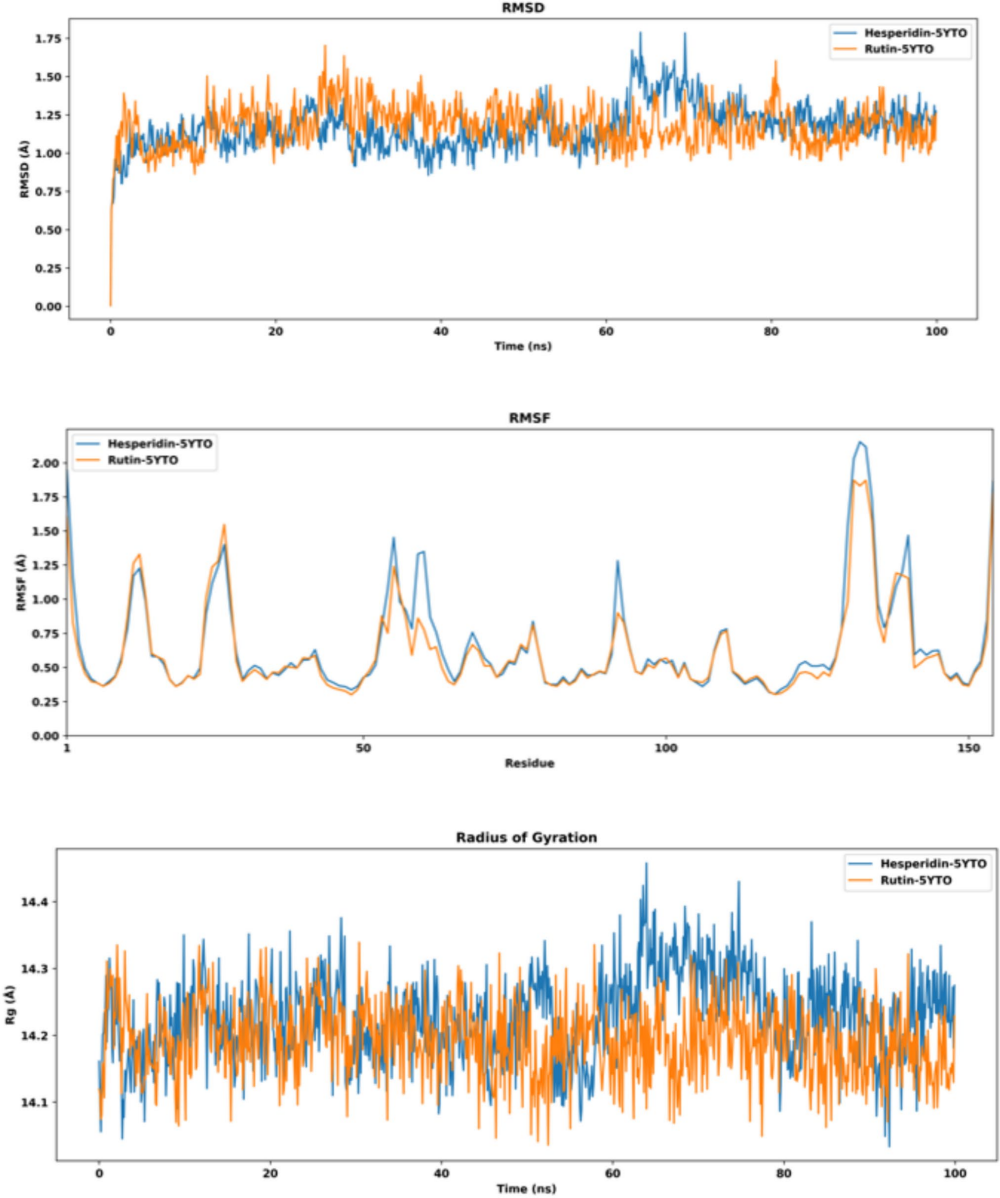
RMSD, RMSF, and RG analysis for the MD simulations of hesperidin-5YTO (blue) and rutin-5YTO (orange) complexes

The highest-ranked phenolics, hesperidin, and rutin, were selected owing to their superior binding affinity scores for SOD-1. Two molecular dynamics simulations were conducted for the hesperidin-5YTO and rutin-5YTO complexes over a timescale of 100 ns. The trajectories were analyzed using RMSD, RMSF, RF, PCA, free energy landscape, and dynamic cross-correlation matrix (DCCM) analyses. The root mean square deviation (RMSD) was calculated to check the conformational stability of SOD1 (PDB ID: 5YTO) protein in complex with hesperidin and rutin over the 100ns simulation equilibrate and is capable of generating a stable structural complex within the RMSD range of 0.9–1.75 Å until 80 ns stimulation. After 80 ns, the complexes stabilized with values 1.25, and 1.15 Å, respectively Fig. 6. Further, root mean square fluctuation (RMSF) analysis determined the residual fluctuation of SOD-1 Proteins in complex with hesperidin and rutin throughout the 100 ns simulation. The SOD-1 Protein conformational remains stable during the total run time of the MD simulation and both complexes are almost identical. The majority of residues across all complexes exhibited low RMSF values ranging from 0.35 to 1.75, indicating complex stability with some fluctuation at some residues from 10–20, 25–35, 50–70, 85–95, and 130–140 for both complexes. Figure 6. The radius of gyration (Rg) demonstrates the compactness of SOD-1 (PDB ID: 5YTO) protein in a complex with hesperidin and rutin over the 100ns simulation. The Rg values are from 14.1 to 14.4 and from 14.1 to 14.3, respectively (Fig. 6). According to the overall Rg results analysis shows that the complexes maintained stable and compact protein structures around the simulation.

**Fig. 7:**
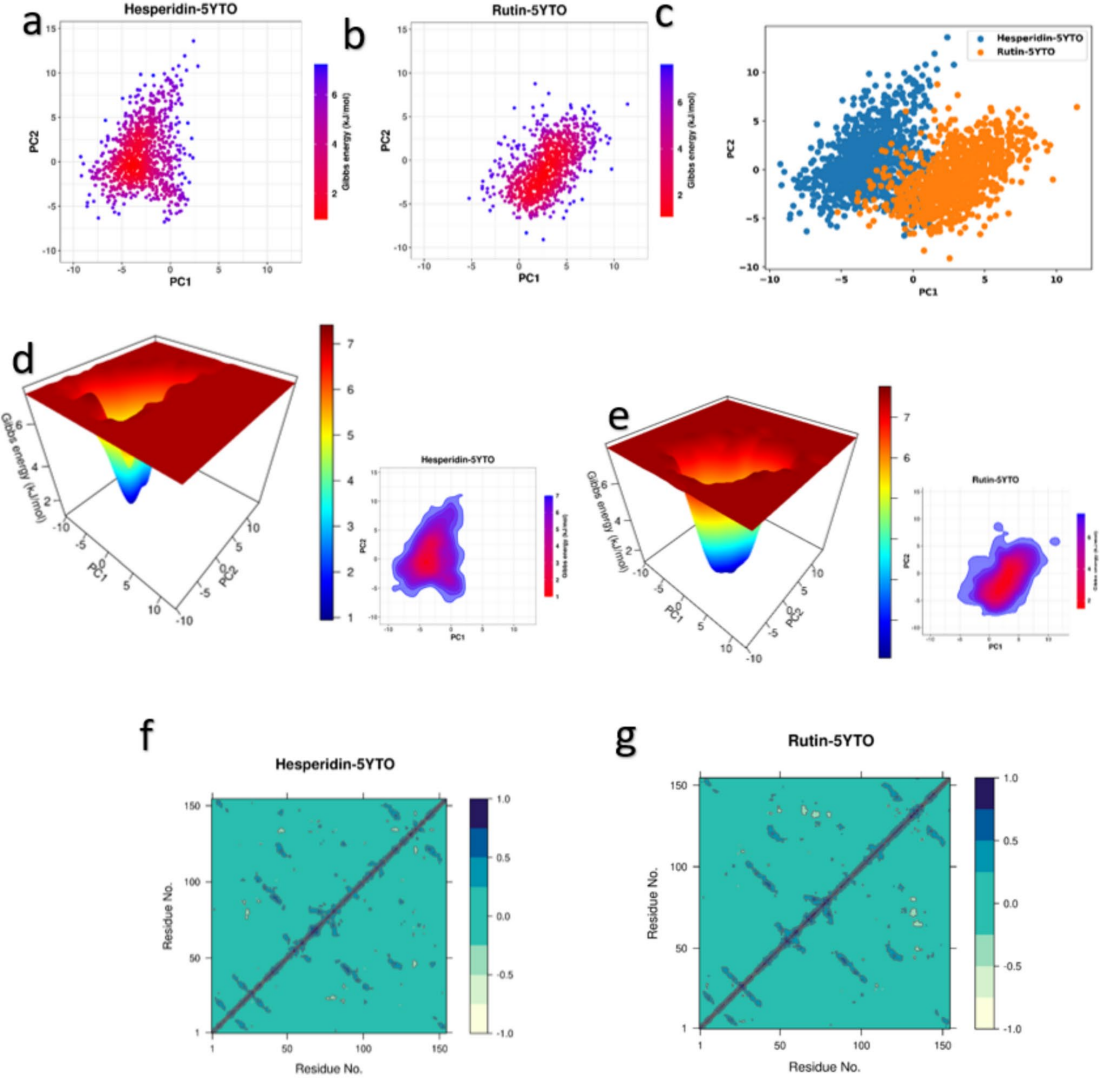
Principal component analysis (PCA), free energy landscape (FEL) and Dynamic cross-correlation map (DCCM) during 100 ns simulation period (**a**) PCA plot of Hesperidin-5YTO complex, (**b**) PCA plot of rutin-5YTO (**c**) Superimposed PCA plot of hesperidin-5YTO and rutin-5YTO complexes (**d**) 2D and 3D (FEL) hesperidin-5YTO complex, and (**e**) 2D and 3D (FEL) rutin-5YTO complex. The color bar denotes the relative free energy value. (**f**) DCCM hesperidin-5YTO complex and (**g**) DCCM rutin-5YTO complex

Principal component analysis (PCA) elucidated and characterized coordinated movements specific to 5YTO protein domains, which influence biological efficiency, affecting system stability and protein functionality. The PCA of the two-stimulation system hesperidin-5YTO and rutin-5YTO complexes (Fig. 7a and b) illustrates the conformational changes during the MD simulations. As the complex systems move and deviate to the right corner and left corner, respectively. The overlaid plot of each two trajectories exhibits a low overlapping, which shows a considerable difference in terms of their conformations (Fig. 7c). This finding suggests that the binding of hesperidin and Rutin plays a crucial role in stabilizing the structural alterations of the two complexes. Subsequently, PCA was employed to conduct a free energy landscape (FEL) analysis to monitor the unique binding conformation and identify the most dominant internal mode of motion through the principal components. Gibbs free energy expresses the global energy minima state. The hesperidin-5YTO and rutin-5YTO complexes (Fig. 7d and e) achieved their lowest free energy (LFE) at 1.8 and 1.7 kJ/mol, respectively. Consequently, the FEL demonstrates that the protein folds to achieve its lowest energy state, which is accurately accomplished due to the ligands'binding. The local energy minimum was demonstrated in the deep blue color region of 3D and the deep red region of 2D, which actively facilitated the stable confirmation. Furthermore, dynamic cross-correlation matrix (DCCM) analysis was performed to understand the conformational motion of amino acid residues of the binding pocket of SOD-1 protein in the presence of Hesperidin and Rutin. The final 10 ns of simulated trajectories were utilized for DCCM analysis. The 2D correlation matrix, ranging from − 1.0 to + 1.0, illustrates multiple levels of correlation between residues, represented by a color gradient from white to dark blue, where values from 0 to + 1 indicate positive correlation and values from 0 to −1 signify negative or anti-correlation. The hesperidin-5YTO and rutin-5YTO complexes with relatively comparable areas represented with dark blue color, which indicate similar residue correlation (Fig. 7f, g). Conclusively, the correlated residues in both complexes support the stability of hesperidin and rutin in the SOD-1 protein active site.

MD simulations confirmed the stability of the top-ranked docked phenolics hesperidin and rutin within the active site of SOD-1 protein, supporting the reliability of our docking results. This aligns with previous studies emphasizing the role of MD simulations in validating molecular docking outcomes in the analysis of bioactive compounds. For instance, a study on *Pongamia pinnata* extract against skin cancer demonstrated that MD simulations were essential in verifying the stability of the complexes EGF-Pazopanib, EGFR-Pongachromene, and ERBB2-Vitexin up to 100 ns without any major fluctuation (Navyatha Karamala et al. 2025). Similarly, research on velvet antler extract against diabetic osteoporosis highlighted the necessity of MD simulations in confirming the stability of binding and interaction of AKT1 to 17-β-estradiol, ATP, Cholesterol, and Estrone that predicted by docking, further reinforcing their significance in phytochemical studies (Wang et al. 2025).

## Conclusion

The AMEs of *A. eggersii*, *C. macrospermum*, and *J. caffra* were notable for their phenolic metabolites, such as flavonoids and phenolic acids. The hepatoprotective results of the three extracts were attributed, at least in part, to their phenolic components which possess active, reducing pharmacophore scaffolds with excellent antioxidants, anti-inflammatory, and anti-apoptotic potential. Extracts have similar phenolic components, yet in variable concentrations, showed comparable hepatoprotective activity. Molecular docking, at least in part, correlates the promising hepatoprotective activity to the good binding affinity of the major flavonoids and phenolic acids in the three extracts to caspase-3, and SOD-1 with possible synergistic effect. Moreover, MD confirms the stability of hesperidin and rutin in the SOD-1 protein active site. Eventually, the AMEs of the three investigated palm tree species were investigated for the first time as protective agents against drug-induced hepatotoxicity, although further clinical investigation is required.

## Supplementary Information

Below is the link to the electronic supplementary material.

Supplementary file1 (DOCX 3.94 MB)

## Data Availability

The data supporting the findings of this study are included within the article and its supplementary materials.

## Abbreviations

*DILI*: Drug-induced liver injury
*AME*: Aqueous methanol abstract
*PCM*: Paracetamol
*MDA*: Malondialdehyde
*CYP450*: Cytochrome P450
*GSH*: Glutathione
*TNF-α*: Tumor necrosis factor-alpha
*IL-1β*: Interleukin 1β
*AST*: Aspartate aminotransferase
*ALT*: Alanine aminotransferase
*GGT*: Gamma-glutamyl transferase
*GST*: Glutathione-S-transferase
*GPx*: Glutathione peroxidase
*SOD*: Superoxide dismutase
*IL-6*: Interleukin 6
*COX-2*: Cyclooxygenase-2
*iNOS*: Inducible nitric oxide synthase
*ROS*: Reactive oxygen species
*MD*: Molecular dynamics
*RMSD*: Root means square deviation
*RMSF*: Root means square fluctuation
*RG*: Radius of Gyration
*PCA*: PCA: Principal Component Analysis
*DCCM*: Dynamic cross correlation matrix
*FEL*: Free energy landscape
