# Supplementary material for: Polyphenolic profile, hepatoprotective evaluation, and molecular docking study of three palm tree species (Family Arecaceae)
Source: Saudi Pharm J. 2025 Jul 8;33(4):21. doi: 10.1007/s44446-025-00017-3 (PMC12238460; doi:10.1007/s44446-025-00017-3)
Supplement: Supplementary file 1 — Supplementary file1 (DOCX 3.94 MB) [file 44446_2025_17_MOESM1_ESM.docx]

**Supplementary tables and figures**

**Table S1.** HPLC–MS/MS applied acquisition parameters (dynamic-MRM mode) for the analysis of the 38 markers

| No. | Compounds | Precursor ion, *m/z* | Product ion, *m/z* | Fragm-entor, V | Collision energy, V | Polarity | Retention time (Rt, min) |
| --- | --- | --- | --- | --- | --- | --- | --- |
| 1 | Gallic acid | 169 | 125.2^*^ | 97 | 12 | Negative | 6.96 |
| 2 | Neochlorogenic acid | 353 | 191.2^*^, 179 | 82 | 12, 12 | Negative | 9.52 |
| 3 | Delphinidin-3-galactoside | 465.01 | 303^*^ | 121 | 20 | Positive | 11.36 |
| 4 | (+)-Catechin | 289 | 245.2^*^,109.2 | 131 | 8, 20 | Negative | 11.44 |
| 5 | Procyanidin B2 | 576.99 | 576.99^*^, 321.2 | 160 | 0, 32 | Negative | 12.41 |
| 6 | Chlorogenic acid | 353 | 191.2^*^, 127.5 | 82 | 12, 20 | Negative | 12.42 |
| 7 | *p*-Hydroxybenzoic acid | 137 | 93.2^*^ | 92 | 16 | Negative | 12.86 |
| 8 | (-)-Epicatechin | 289 | 245.1^*^, 109.1 | 126 | 8, 20 | Negative | 13.03 |
| 9 | Cyanidin-3-glucoside | 449 | 287.3^*^, 255.6 | 121 | 20, 20 | Positive | 13.14 |
| 10 | Petunidin-3-glucoside | 479.01 | 317^*^, 302 | 121 | 20, 44 | Positive | 13.26 |
| 11 | 3-Hydroxybenzoic acid | 137 | 93.2^*^ | 88 | 8 | Negative | 13.59 |
| 12 | Caffeic acid | 179 | 135.2^*^, 134.1 | 92 | 12, 24 | Negative | 13.65 |
| 13 | Vanillic acid | 167 | 152.4^*^, 108.1 | 88 | 12, 20 | Negative | 14.32 |
| 14 | Resveratrol | 227 | 185^*^, 143.2 | 136 | 12, 20 | Negative | 14.40 |
| 15 | Pelargonidin-3-glucoside | 433.01 | 271^*^, 121 | 116 | 24, 50 | Positive | 14.52 |
| 16 | Pelagonidin-3-rutinoside | 579.01 | 271^*^ | 145 | 32 | Positive | 14.56 |
| 17 | Malvidin-3-galactoside | 493.01 | 331^*^, 315.1 | 121 | 20, 50 | Positive | 14.64 |
| 18 | Syringic acid | 196.9 | 182.2^*^, 121.2 | 93 | 8, 12 | Negative | 15.28 |
| 19 | Procyanidin A2 | 575 | 575^*^, 285 | 170 | 0, 20 | Negative | 16.18 |
| 20 | *p*-Coumaric acid | 163 | 119.2^*^, 93.2 | 83 | 12, 36 | Negative | 16.70 |
| 21 | Ferulic acid | 193 | 134.2^*^, 131.6 | 83 | 12, 8 | Negative | 17.10 |
| 22 | 3,5-Dicaffeoylquinic acid | 514.9 | 353.1^*^, 191 | 117 | 8, 28 | Negative | 17.61 |
| 23 | Rutin | 609 | 300.2^*^, 271.2 | 170 | 32, 50 | Negative | 17.73 |
| 24 | Hyperoside | 465.01 | 303^*^, 61.1 | 97 | 8, 50 | Positive | 18.33 |
| 25 | Isoquercitrin | 463 | 271.2^*^, 300.2 | 155 | 44, 24 | Negative | 18.36 |
| 26 | Delphinidin-3,5-diglucoside | 462.9 | 300.1^*^ | 165 | 24 | Negative | 18.38 |
| 27 | Phloridzin | 435.39 | 273^*^, 167 | 155 | 8, 28 | Negative | 18.83 |
| 28 | Quercitrin | 446.99 | 300.2^*^, 301.2 | 160 | 24, 16 | Negative | 19.61 |
| 29 | Myricetin | 316.99 | 179.1^*^, 182 | 150 | 16, 24 | Negative | 19.61 |
| 30 | Naringin | 578.99 | 271.3^*^, 151.3 | 170 | 32, 44 | Negative | 19.62 |
| 31 | Kaempferol-3-glucoside | 447 | 284.2^*^, 255.2 | 170 | 24, 40 | Negative | 19.77 |
| 32 | Hesperidin | 611.01 | 303^*^, 334.8 | 112 | 20, 12 | Positive | 20.19 |
| 33 | Ellagic acid | 301 | 301^*^, 229 | 170 | 0, 24 | Negative | 21.41 |
| 34 | *Trans*-Cinnamic acid | 149 | 131.2^*^, 77.2 | 74 | 4, 36 | Positive | 21.44 |
| 35 | Quercetin | 300.99 | 151.2^*^, 179.2 | 145 | 16, 12 | Negative | 21.87 |
| 36 | Phloretin | 272.99 | 167^*^, 123 | 116 | 8, 20 | Negative | 22.30 |
| 37 | Kaempferol | 287.01 | 153^*^, 69.1 | 60 | 36, 50 | Positive | 23.84 |
| 38 | Isorhamnetin | 314.99 | 300.2^*^, 196.1 | 145 | 16, 4 | Negative | 24.57 |

^*^ These product ions were used for quantification.

**(A)**

**(B)**

**Figure S1:** HPLC-MS/MS profile **(A)** Total Ion Chromatogram acquired in negative ion mode for *A. eggersii*; **(B)** Total Ion Chromatogram acquired in positive ion mode for *A. eggersii*

**(A)**

**(B)**

**Figure S2:** HPLC-MS/MS profile **(A)** Total Ion Chromatogram acquired in negative ion mode for *C. macrospermum*; **(B)** Total Ion Chromatogram acquired in positive ion mode for crude *C. macrospermum*

**(A)**

**(B)**

**Figure S3:** HPLC-MS/MS profile **(A)** Total Ion Chromatogram acquired in negative ion mode for J.caffra; **(B)** Total Ion Chromatogram acquired in positive ion mode for J.caffra

| **3D Visualization** | **2D Visualization** |
| --- | --- |
| 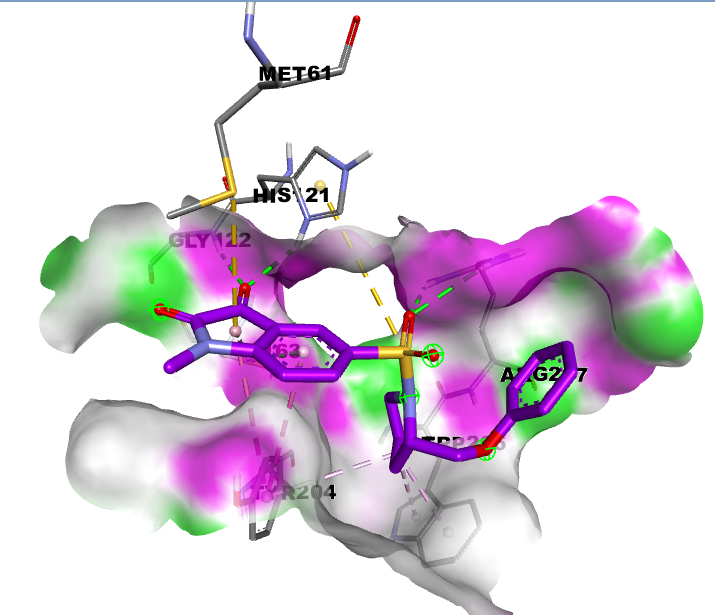 | 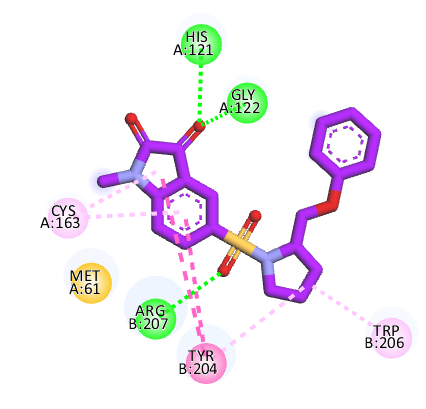 |
| **Crystallized ligand** (PDB ID: MSI) | |
| **Phenolic acids** | |
| 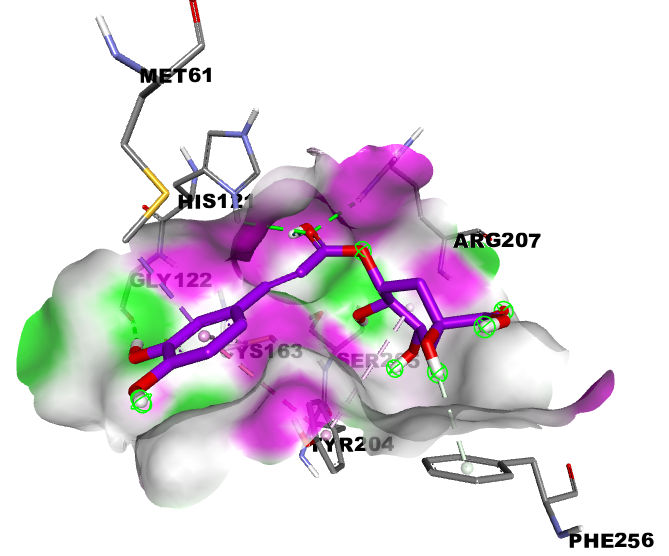 | 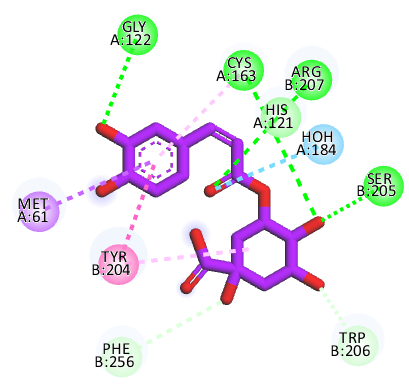 |
| Compound 1 (Chlorogenic acid)_ | |
| 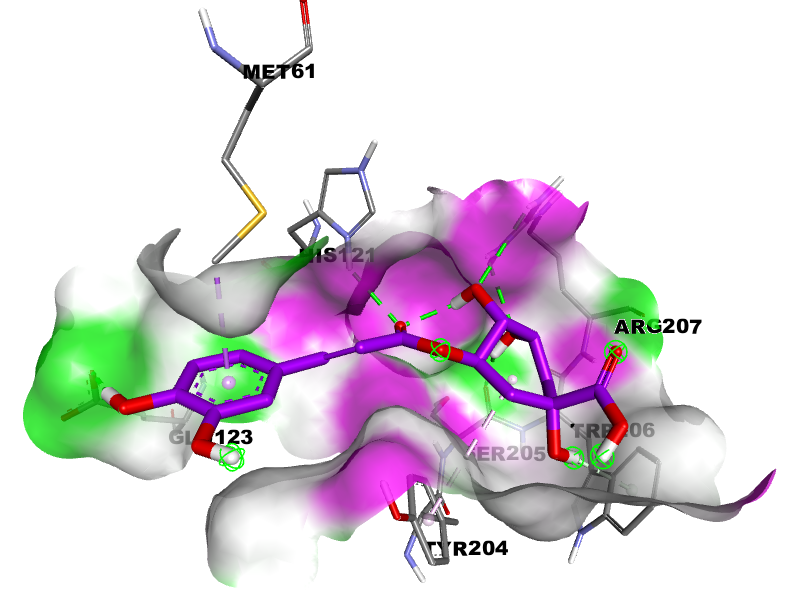 | 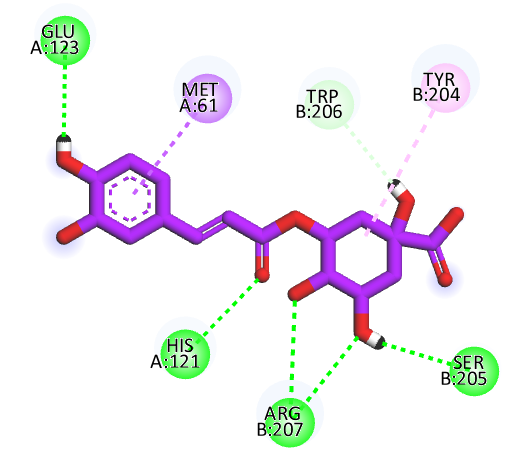 |
| Compound 2 (Neochlorogenic acid)_ | |
| 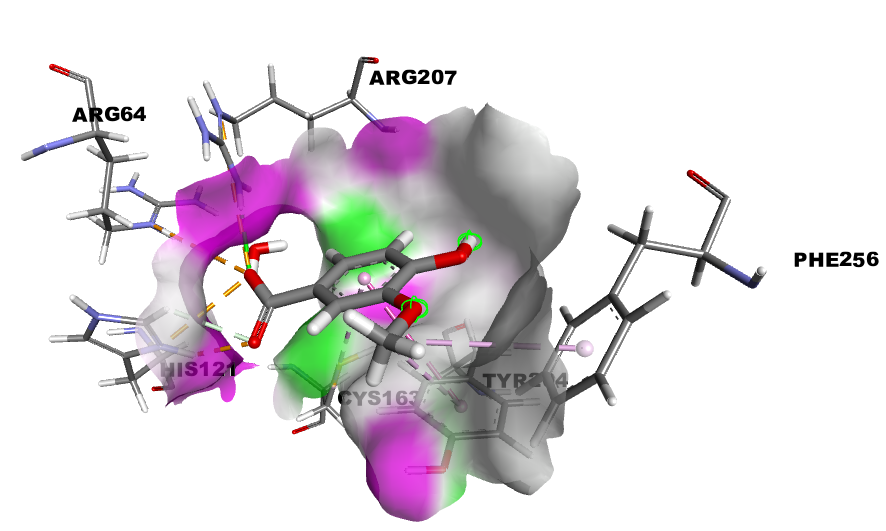 | 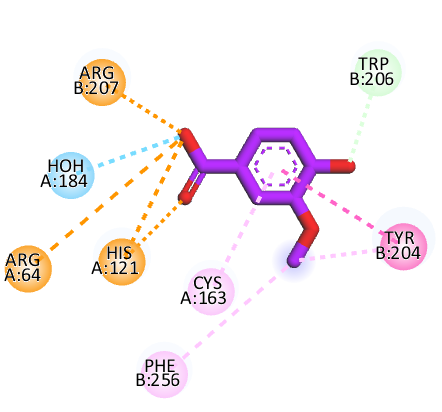 |
| Compound 3 (Vanillic acid) | |
| 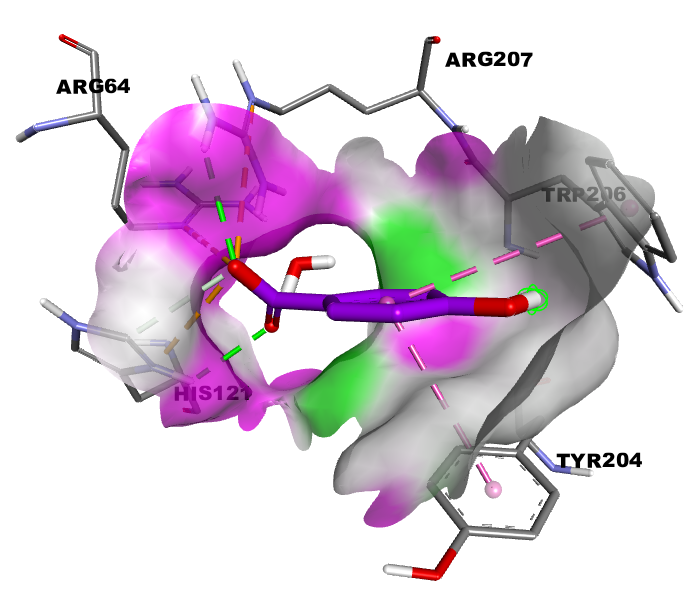 | 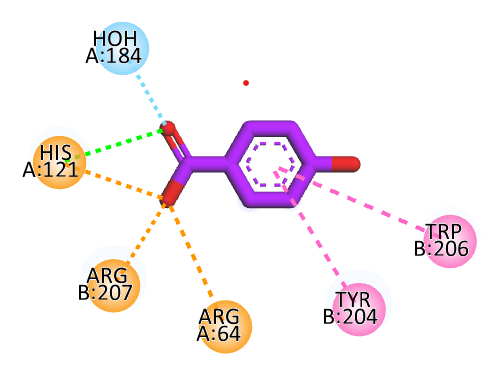 |
| Compound 4 (p-Hydroxybenzoic acid) | |
| **Flavonols** | |
| 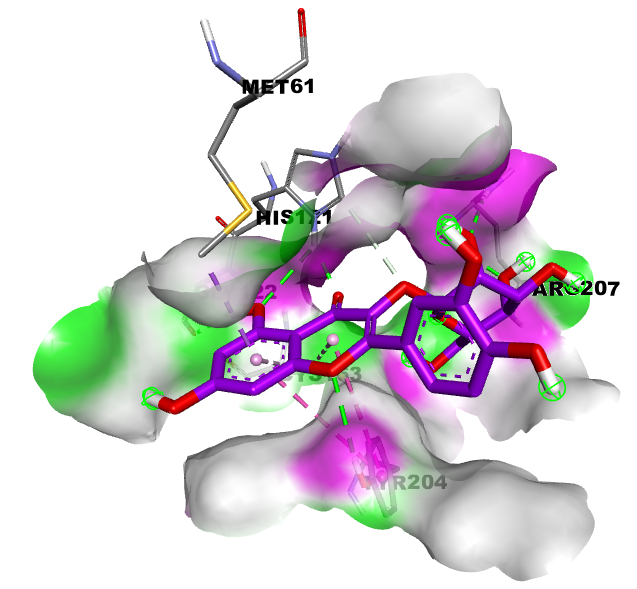 | 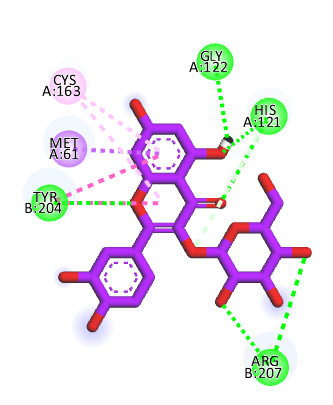 |
| Compound 6 (Hyperoside) | |
| 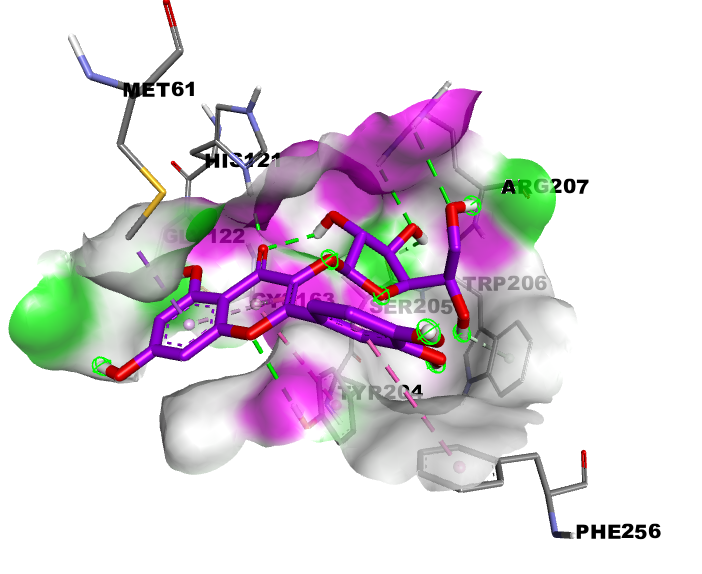 | 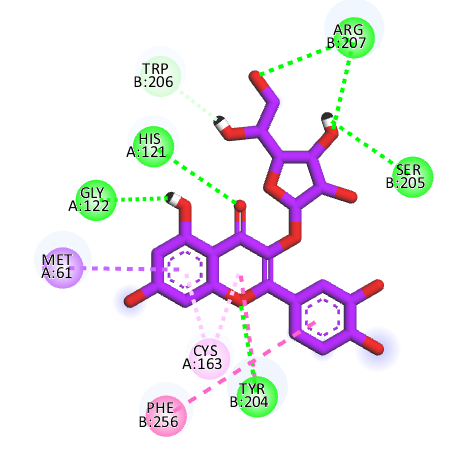 |
| Compound 7 (Isoquercitrin) | |
| **Flavan-3-ols** | |
| **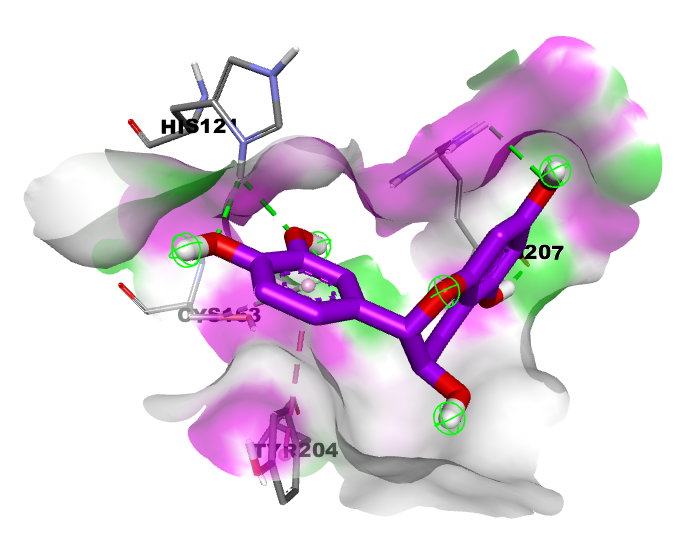** | 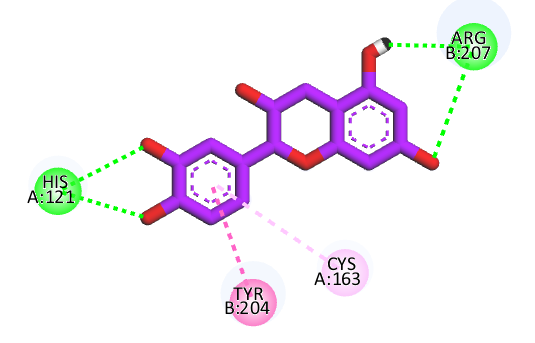 |
| **Compound 9 (**Catechin) | |

**Figure S4:** 3D (Left) and 2D (Right) structure interaction poses of caspase-3 (PDB ID: 1GFW) with Co-crystallized ligand (an isatin sulfonamide inhibitor) and the major identified phenolic compounds in the AMEs of *A. eggersii, C. macrospermum,* and *J.caffra* .

| **3D Visualization** | **2D Visualization** |
| --- | --- |
| 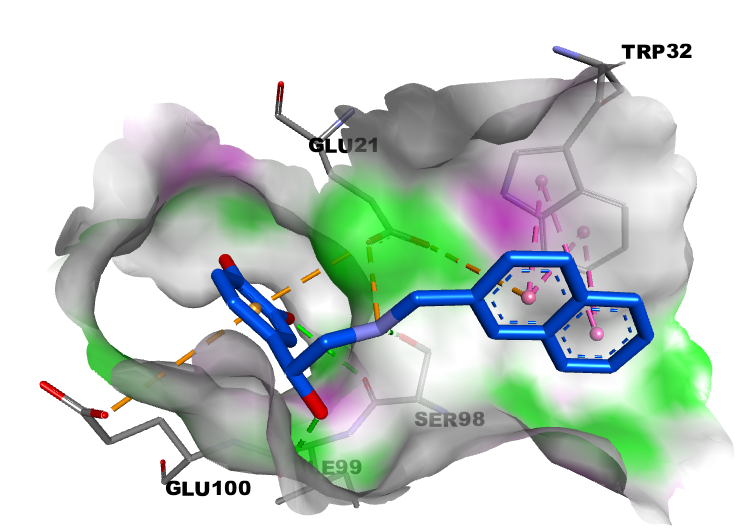 | 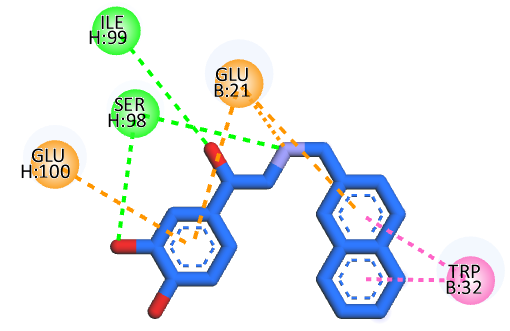 |
| **Crystallized ligand** (PDB ID: 946) | |
| **Phenolic acids** | |
| 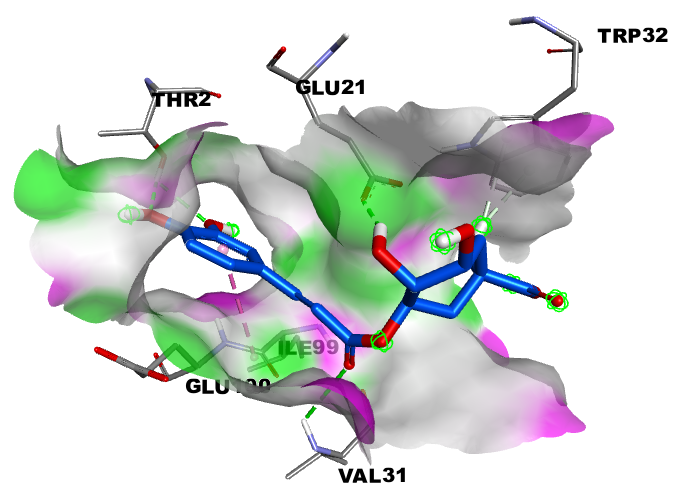 | 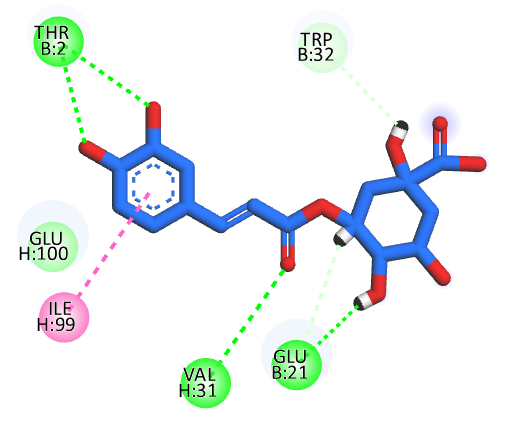 |
| Compound 1 (Chlorogenic acid)_ | |
| 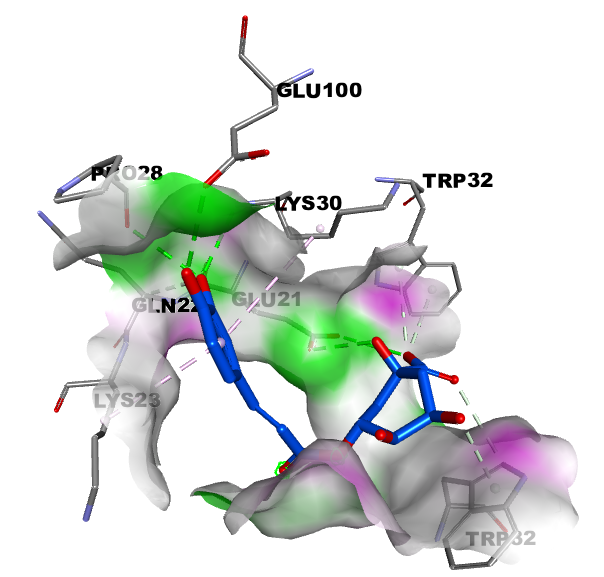 | 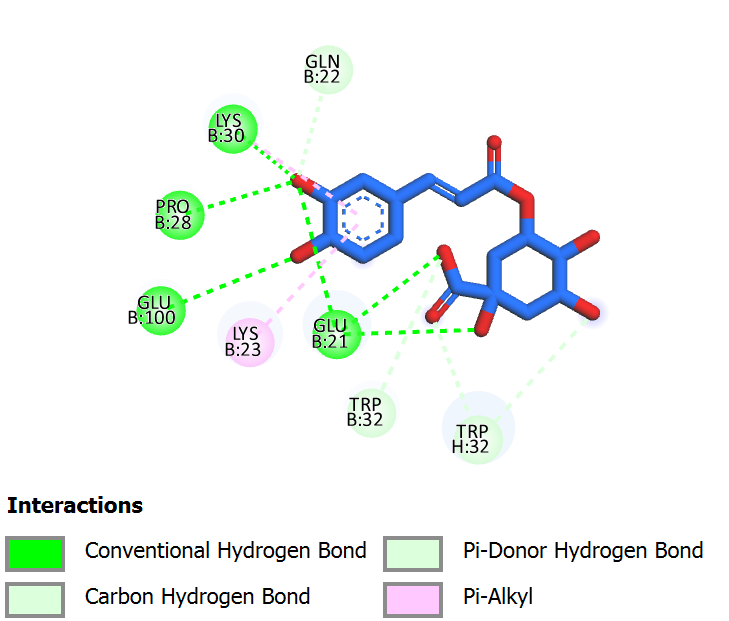 |
| Compound 2 (Neochlorogenic acid)_ | |
| 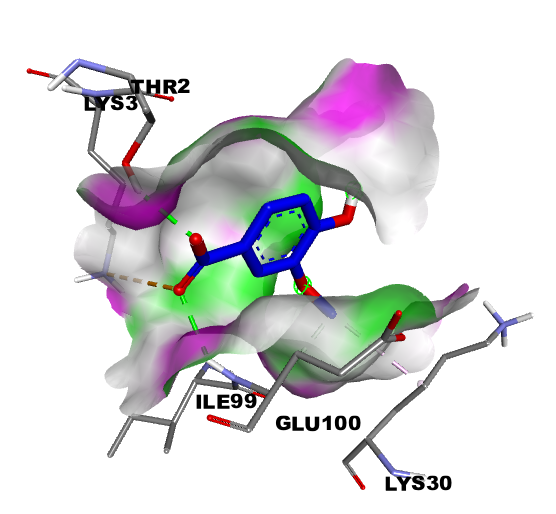 | 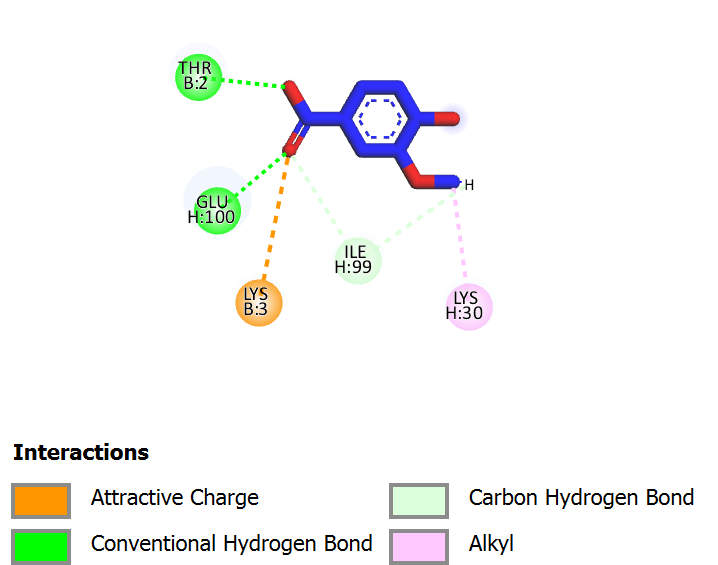 |
| Compound 3 (Vanillic acid) | |
| 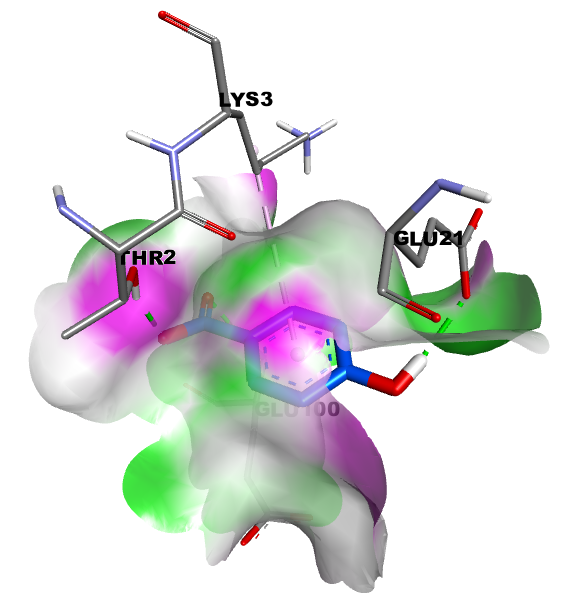 | 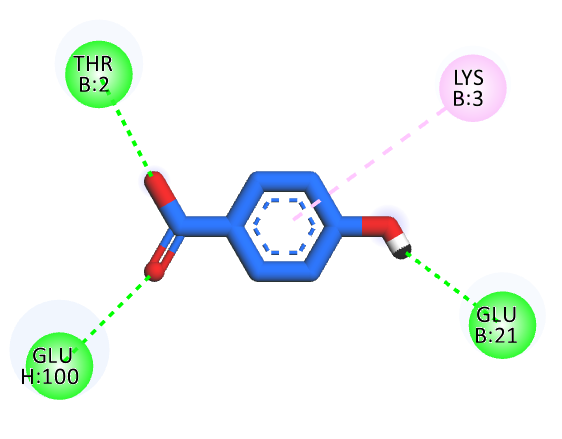 |
| Compound 4 (p-Hydroxybenzoic acid) | |
| **Flavonols** | |
| 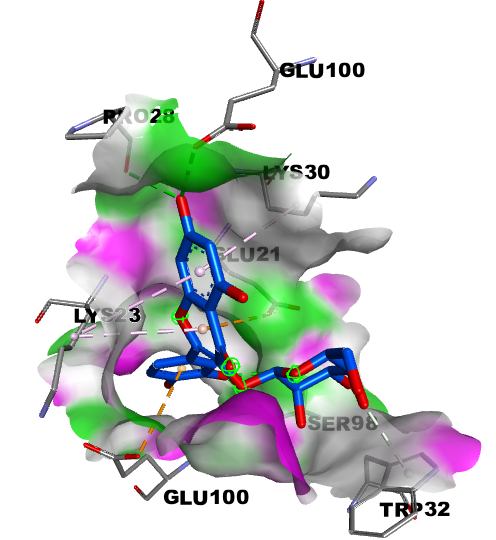 | 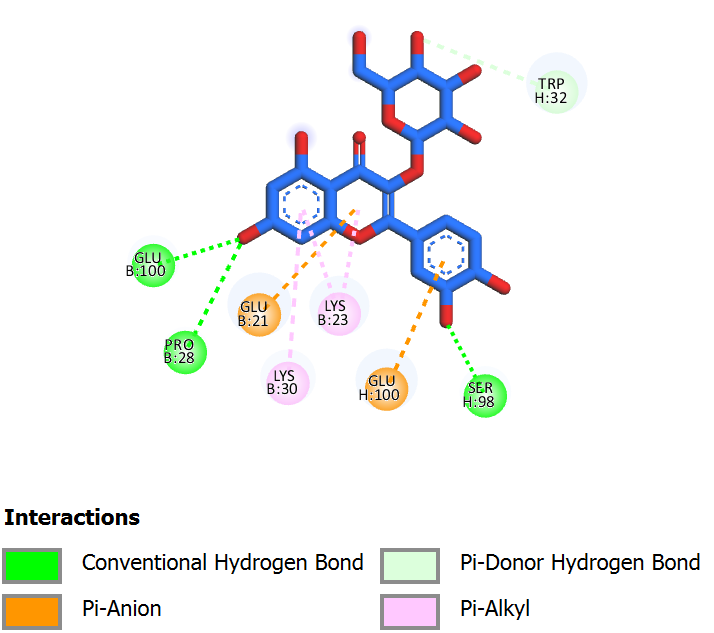 |
| Compound 6 (Hyperoside) | |
| 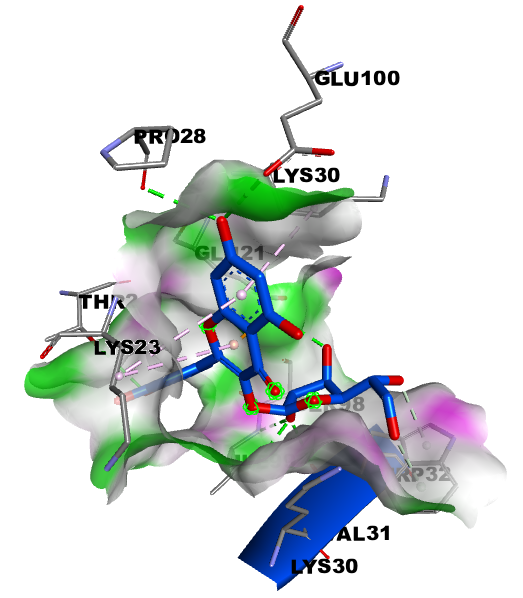 | 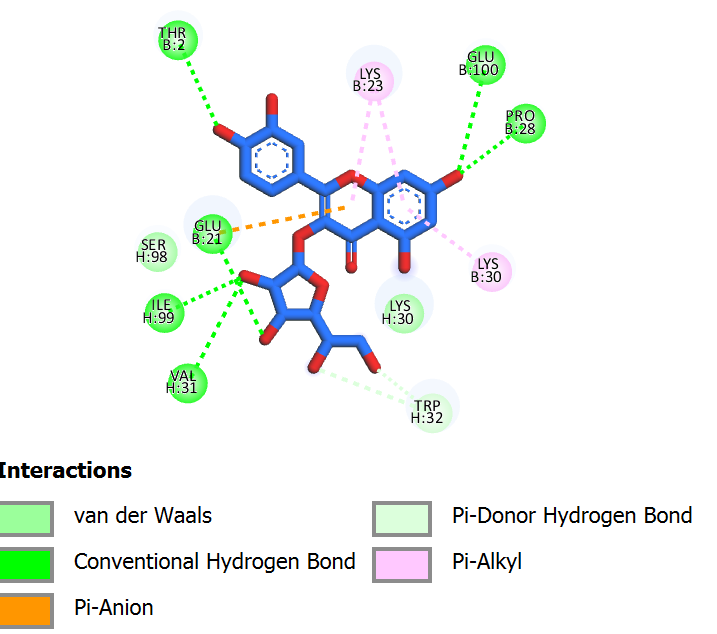 |
| Compound 7 (Isoquercitrin) | |

**Figure S5:** 3D (Left) and 2D (Right) structure interaction poses of SOD-1 (PDB ID: 5YTO) with Co-crystallized ligand (naphthalene-catechol linked compound) and the major identified phenolic compounds in the AMEs of *A. eggersii, C. macrospermum,* and *J. caffra*.
